# Mapping community pathways to employment for youth with disabilities—a realist review

**DOI:** 10.3389/fpubh.2026.1743478

**Published:** 2026-03-17

**Authors:** Amelia Hagelstam-Renshaw, Roberta Cardoso, Jinan Zeidan, Ebrahim Mahmoudi, Linda Nguyen, Eleni Philippopoulos, Keiko Shikako

**Affiliations:** 1 Participation and Knowledge translation in Childhood Disability Collaborative, School of Physical and Occupational Therapy, McGill University, Montreal, QC, Canada; 2 Faculty of Social Work, University of Calgary, Calgary, AB, Canada; 3 Schulich Library of Physical Sciences, Life Sciences and Engineering, McGill University, Montreal, QC, Canada

**Keywords:** community factors, disability, employment, intersectionality, realist review, school-to-work, transition, youth

## Abstract

**Background and objectives:**

Youth with disabilities face significant challenges in accessing employment and transitioning from school-to-work settings. Youth in intersectional contexts face additional barriers. The ecological development theory posits that macro factors such as policies, programs, universal design, and accessible community infrastructure may foster youth participation across sectors and create pathways to employment. This review aimed: (a) to identify the community-based factors related to the employment of youth ages 15–25 with all types of disabilities, and (b) describe the contexts and mechanisms within employment readiness programs that contribute to successful school-to-work employment outcomes.

**Methods:**

We conducted a realist review following RAMESES standards on Medline, PsycINFO, ERIC, Policy Commons, Sociological Index, Google Scholar, REHABData and Canadian Research Index. Search terms were related to employment, disabilities, youth, and community factors. Analysis was grounded in the Ecological Systems Theory. The Child Community Health Inclusion Index (CHILD-CHII) Macro-Level factors were used as a framework for data extraction and analysis, including transportation, staff training, community design, awareness initiatives, healthcare access, general programs/services, volunteer/work, education, technology, web-mapping, accessibility policies, and social factors. The Gender Based Analysis Plus (GBA+) Framework was used to explore intersectionality in the programs described. Data was grouped into context, mechanisms, and outcomes to provide an overview of what programs exist, in what circumstances and what type of outcomes are considered successful employment in the existing research.

**Results:**

From 13,167 studies identified, 56 articles met the inclusion criteria. The most prominent Mechanisms included: education (*n* = 25) such as skills training (social, Cv writing, etc.) or providing information on post high school education options, and general programs/services (*n* = 38) such as job search or readiness training, and mentorship programs. Successful transition involved skills building, providing social supports, and increasing collaboration and communication between school and workspaces. Negative findings included skills learnt in program settings not translating to real-world settings, services for youth ending after program completion, a failure of coordination between community systems, and structural barriers in the workplace (youth dealing with pain management/fatigue). No considerations of intersectionality were addressed in the majority of these studies.

**Discussion:**

Existing youth employment programs focus mainly on the micro environment aspects and individual skills training, job preparedness and individual/employer-targeted strategies. Few mechanisms addressing the meso environment include socials supports and some limited services; however, the macro context of development is rarely addressed, with policies, standards, regulations and systems-levels programs that are not designed to favor employment outcomes for youth with disabilities, particularly those with intersectional realities. Understanding community ecologies, and system-level determinants of employment is critical to the development of inclusive communities and equitable employment opportunities.

## 1 Introduction

Youth with disabilities face significant challenges in accessing employment spaces and transitioning from a school setting to a work environment. In Canada, in 2022, the employment rate for youth with disabilities from the ages of 15 to 25 was at 54.4% compared to 64.4% for peers of the same age without disabilities (1). For the purpose of this review we adopt the International Labor Organization definition of employment as an “activity to produce goods or services in exchange for remuneration” (2). This includes short-term employment opportunities such as paid internships and apprenticeship, and long-term employment opportunities, including contract work and permanent positions. This definition excludes unpaid internships, apprenticeships or other opportunities that are without financial compensation. In 2024, the government of Canada launched standards for employment of persons with disability (3). Although standards are developed to address the macro and meso contexts where employment occurs, they do not address the specific contexts where youth grow and seek employment, and particular barriers to employment for young people with disabilities who are also part of other groups such as girls, women or ethnic minorities (4). The existing supports for employment may occur in the meso environment of youth, in systems such as schools or rehabilitation settings, which may not consistently provide students with disabilities with the tools and skill sets that they might need to thrive and succeed in a work environment (5). The opportunities for informal job opportunities, social interactions and overall participation in the community may be limited for young people with diverse disabilities.

Bronfenbrenner's Ecological Systems Theory (6) posits that individuals are shaped by a multitude of interconnected systems within their surroundings. The Microsystem, the Mesosystem, the Exosystem, the Macrosystem and the Chronosystem interact and mutually influence each other as the individual develops in a given societal context. Directly related to the individual is the Microsystem—an individual's immediate surrounding such as their family or friends, and the Mesosystem—the immediate outwards connections of an individual such as extra-curricular activities of an individual's family. Further from the individual is the Exosystem, relating to structures such as governments or family friends, and the Macrosystem, relating to established social attitudes such as norms, values and cultural ideologies. Lastly, at the largest level, this theory proposes the Chronosystems—an individual's environment over an extended period of time (6). Within the contexts of youth employment, we consider that the micro, meso, and macro systems influence youth's development, well-being, and transition into adulthood, contributing to shape experiences that may lead to employment outcomes. Social norms, attitudes or cultural values vary across contexts, and may result in different outcomes. The contexts that surround a youth with a disability, and the contexts that surround youth transition programs, influence the program itself as well as the effects it has on its participants. The cultural norms and values or Macrosystem of the environment in which the program runs, will affect the same program differently, running in a different environment with a different Macrosystem. Within the systems proposed by Bronfenbrenner, it is not only the effects of the individual systems independently shaping the individual, but the complex interconnectedness of the ecological model interacting with oneself, creating a unique set of contexts that contribute to the youth's environment. This also changes over time and during the youth growth trajectory, hence the chronosystem will impact, in the long term, a community's ability to include youth and create employment opportunities. For instance, aspects such as access to appropriate educational supports and health services, supportive same-age peers and adults are known to be fundamental to supporting youth in successfully integrating workplaces (7). Additionally, support from vocational counselors, educators or teachers in finding and maintaining employment has been shown to be a key component of many vocational support programs for youth (8). The transition from school-to-work is a key period for youth development and well-being. The early work experiences play a large role in shaping youth plans for the future. They allow youth to explore their interests and strengths, gain skills for the workforce, and allowing them to build connections within their communities. At the macro level, policies, laws, standards, and regulations can create the right conditions at the societal level for the creation of employment opportunities and inclusive systems that respect international laws and conventions.

To navigate the complexity of these interconnected systems and their influence on employment outcomes, this study adopts a realist review methodology (9). This approach was chosen over a traditional systematic review due to its unique suitability for studying complex social interventions like employment support programs (10), as it seeks to answer not just if programs work, but “what works, for whom, in what circumstances, and why?” (10). The strengths of this approach include its ability to synthesize diverse evidence types (qualitative, quantitative, mixed-methods) and its focus on generating explanatory theories about how context and mechanisms interact to produce outcomes (9).

A general understanding exists about key elements needed to support employment pathways for youth with disabilities such as, early school-based learning, workforce preparation experiences, or opportunities for youth to develop their leadership skills and connect with peers (11). Nevertheless, the existing literature presents a limited description of the broad contextual and community characteristics of available systems and programs supporting the school to work transition. Examples of community and contextual factors include transportation to and from employment preparation activities and the capacity building on use of technology beyond strict employment training.

Current literature often describes programs focusing on one type of disability (12) (Supplementary Table 1), or one approach to training such as mentorship or skills training (13), and in specific contexts such as school (14) or rehabilitation/hospital settings (15). A detailed understanding of the different mechanisms adopted in different programs, and the contexts where they occur may allow for a broader application and generalization of successful practices. This knowledge can, in turn, inform macro factors such as the development of policies and standards for employment practices that can in turn be reflected within meso and micro contexts.

This review aims to answer the following research question: What are the community-based factors (context) that are considered in programs (mechanisms) contributing to the employment preparation (outcome) of youth with disabilities? The specific objectives of this review are to (a) identify the community-based factors that contribute to the employment of youth with disabilities, and (b) identify employment pathways to inform the development of inclusive employment standards for youth with disabilities.

By conducting a realist review we will describe research on a multitude of programs that have incorporated different community factors within their curriculums, across multiple landscapes and settings, considering many types of disabilities. We expect to inform policy and standards development that consider the factors contributing to creating ecologies around young people with disabilities to successfully access vocational spaces, mapping employment pathways for this equity-deserving group.

## 2 Methods

A realist review was conducted to identify the context, mechanisms and outcomes described in peer-reviewed research literature on community programs that support employment for youth with disabilities. We followed the Realist And Meta-narrative Evidence Syntheses: Evolving Standards (RAMESES) publication standards (9) for realist review reporting.

### 2.1 Theoretical underpinnings informing the realist review

Our review is theoretically grounded in two key frameworks: Bronfenbrenner's Ecological Systems Theory (6) and GBA+ Framework (16). This dual-framework approach was chosen to build a comprehensive initial program theory that accounts for both the multi-layered environmental influences on youth development and the overlapping systems of discrimination that create unique experiences for youth when transitioning across systems of care and social structures. Intersectionality theory guides the understanding that the pathways toward employment are impacted by Bronfenbrenner's micro factors operationalized through individual identities: women, men, and gender-diverse groups, and the “plus” in the GBA+ tool including identity factors such as race, age, and disability. Based on principles of substantive equality, we aimed at understanding how youth employment programs address the different identities of youth and how communities consider or neglect youth's intersectional identities as they navigate through the macro and exo systems leading to employment.

We anchor our analysis on two frameworks that operationalize the program theories:

1. The Child Community Health Inclusion Index (CHILD-CHII) (17) a framework that was developed based on the ecological model of development and normative human rights frameworks that ground the concepts of substantive equality. Applied within community settings, this tool is comprised of an assessment that evaluates the program itself (Organizational Assessment), the infrastructure in which the program operates (On-Site Assessment), and the policies and external factors within the community where the program is held that affect the program (Macro-Level factors). The specific Macro-Level factors are transportation, staff training, community design, awareness initiatives, healthcare access, general programs/services, volunteer/work, education, technology, web-mapping, accessibility policies, and social factors. We adopted these frameworks to guide our search, data extraction and synthesis, and to support operationalizing, testing, and refining the theoretical concepts.
2. The GBA+ framework provides structured lenses to understand the complexities of social inequality, underscores the importance of addressing the intersecting systems through which it is perpetuated (18) and highlights structural barriers variable based on interconnecting social positions (19). “In hidden processes of oppression” (20). Despite these limitations, intersectionality was selected for this study due to its capacity to emphasize structural obstacles experienced by youth with disabilities with intersecting social identities. The distinctions between sex and gender were established in consultation with partners (Disabled Women Network Canada), following a realist approach. Based on the definitions from GBA+ Framework (21), sex was defined as the biological attributes within human and animals, associated with physical anatomical features, gene composition, and hormone levels and functions (21). Based on the same analysis tool, we defined gender as socially constructed, forming social roles and personal identities and influencing personal perception, power distributions, and economic outcomes (21).

Based on these frameworks, our initial program theory posited that: Successful employment transitions for youth with disabilities (Outcome) happen in a combination of program-level structures (meso context) and individual-level (micro context) training (Mechanism), but are contingent upon a supportive and interconnected ecological system (Context). This system includes enabling factors at the meso-level (e.g., strong school-employer partnerships) and macro-level (e.g., inclusive policies, accessible transportation, positive social attitudes). Furthermore, we theorized that the effectiveness of these mechanisms is moderated by the youth's intersectional identity, with those facing multiple forms of marginalization requiring more tailored or intensive supports to achieve equitable outcomes (20–23).

### 2.2 Search strategy

We developed a comprehensive search strategy with the support of an experienced librarian (EP). The search strategy included four main groups of keywords that included terms relating to: employment, youth, disability, and terms referring to the specific CHILD-CHII Macro-Level community factors (for a complete list of search terms refer to the Supplementary Figure 1). We searched the following databases on April 25, 2024: Medline (OVID), PsycINFO (OVID), ERIC (EBSCOhost), Policy Commons (Coherent Digital), Sociological Abstracts (ProQuest), REHABData and Canadian Research Index (ProQuest). These databases were chosen based on the descriptive nature of realist reviews and the broad scope of the sectors included in the larger “community”, spanning from health and social sciences, in which employment programs for youth with disabilities can occur. A search was conducted in Google Scholar and the first 200 results were extracted to identify any literature published outside traditional databases. We also screened the references of the included articles to find additional relevant literature that may have been missed in the initial database searches. No search filters related to age or geographic location were created, however a publication date limit was applied, and only articles published from 2008 onwards were included. The full search strategy is available for consultation in Supplementary file 1.

#### 2.2.1 Inclusion and exclusion criteria

We included peer-reviewed articles published between January 2008 and April 2024. These dates correspond to the signing of the United Nations Convention on the Right of Persons with Disabilities (24), which we expected to be guiding macro factor informing the development of inclusive communities. We included publications: (1) in English or in French, which are the official languages of Canada, (2) reporting on multiple disability groups, focusing on developmental disabilities including: Intellectual and learning disabilities, physical disabilities, autism, visual, deaf and hard-of-hearing, behavioral and mental health conditions. We focused on these disability groups because they are most commonly represented in the youth transition literature and in school-to-work programs. We acknowledge that this focus excludes other important disability groups, such as youth with traumatic brain injuries or other acquired disabilities, which represents a limitation of this review. Future research should examine employment pathways for these underrepresented groups. (3) Involving youth ages 15–25 years old, and (4) focusing on school-to-work transition programs.

### 2.3 Data screening

**Figure 1:**
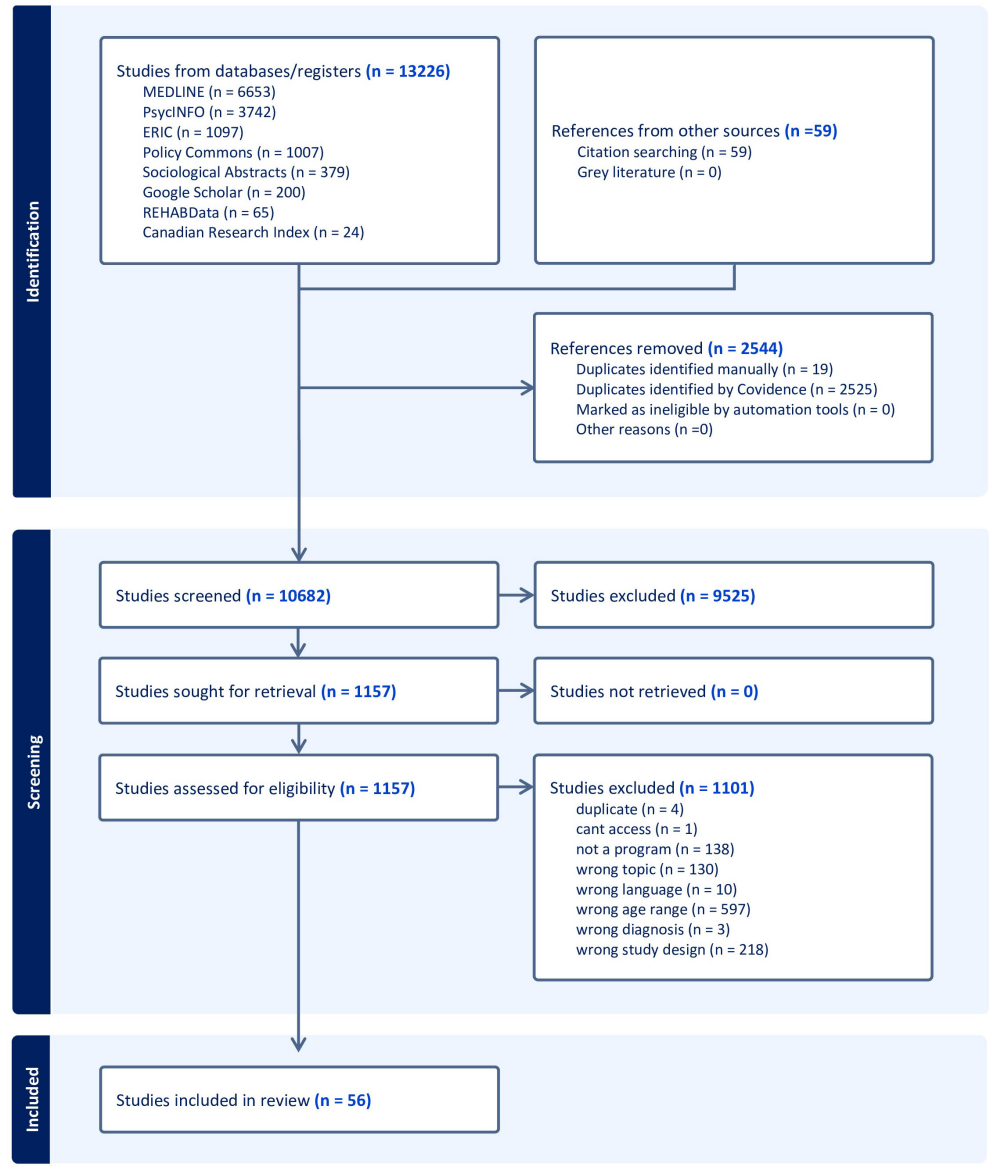
PRISMA flow diagram of study selection.

After an initial review of 10,682 titles, we refined the scope of research to employment programs as opposed to the initial broader scope of school-to-work transition literature, for feasibility purposes and to maintain the Context-Mechanism-Outcome (C-M-O) structure clearer. Initially, 1,157 articles met the inclusion criteria and were screened for full text, after which 56 articles were retained for data extraction and analysis (see Figure 1 for flow chart).

Title and abstract were screened by dyads of authors (AH-R and one other co-author) independently, using Covidence software (22) for inter-rater reliability in selection. Multiple pilot screening sets with 10–50 articles were performed, seeking an inter-reliability rate of at least 90% before proceeding to screening the entire list of titles, abstracts, and full text. Blind selection disagreements identified in Covidence during the inclusion and exclusion process were resolved by a third author (RC). Uncertainties in inclusion and exclusion and data extraction were resolved in regular discussions with the senior author (KS). Throughout this process, reviewers met regularly to discuss any differences in screening, and to discuss any questions that arose.

### 2.4 Data extraction, analysis, and theory refinement

Data extraction was guided by our initial programs theory. We developed a data extraction template that included the CHILD-CHII Macro-Community at Large Factors and GBA+ tool factors (see Supplementary Table 1 for data extraction sheet) and identified C-M-O structures. We extracted all relevant data from the included articles, coding for context, mechanisms, and outcomes. We then formulated CMO hypotheses for each study, identifying how specific contextual factors enabled or constrained certain mechanisms to produce the observed outcomes. The process of identifying mechanisms was theory-driven and interpretive.

Following the principles of realist synthesis, we distinguished mechanisms (the underlying processes or resources that generate change, such as “building self-efficacy” or “fostering social capital”) from program activities or strategies (the practical interventions used, such as “mentorship sessions” or “CV workshops”). For example, a mentorship program (activity) might trigger a mechanism of ‘increased social capital' which, in the context of a supportive employer, leads to a job offer (outcome). We synthesized these CMOs across studies, looking for demi-regularities or patterns. This involved identifying recurring CMO configurations and noting where, why, and for whom they appeared to be most effective. We paid particular attention to evidence that contradicted our initial theory, such as instances of program failure or negative outcomes, as these are crucial for refining the theory. Through this synthesis, we elaborated on our initial program theory, developing a more nuanced, middle-range theory that explains the complex pathways to employment for youth with disabilities. The analysis was an iterative process of testing and refining our initial program theory in close collaboration and consultation with an advisory group constituted of youth with disabilities. Through screening, data extraction, and analysis processes, multiple pilots were performed during the data extraction phase until 90% intrareliability rate was achieved among dyads of authors conducting the data extraction. Once 90% reliability was established, one reviewer (AH-R) extracted data for all articles, with a second reviewer checking the extracted data for reliability.

## 3 Results

Figure 1 shows the review flow diagram and Table 1 presents the definitions of the CHILD-CHII domains as operationalized in the research articles retained for analysis. Most factors identified related to the meso environment, particularly programs and services (*n* = 38), education (*n* = 25) and social aspects (*n* = 14). Table 2 offers the complete list of articles retained, organized by CHILD-CHII Factors. The research identified presented programs acting mostly on micro and meso systems, such as offering opportunities for social gatherings and mentorship paired with job placements as well as specific skills training. Many programs were held within school settings and hospitals, but studies presented limited exploration of how the exosystems such as policies and funding contributed to the implementation, development and configuration of youth employment programs. Table 3 provides the C-M-O structures of the included studies. For in-depth information on the C-M-Os of the programs and their links to Bronfenbrenner's Ecological Systems Theory in our initial programme theory, see Supplementary Table 2. The realist analytical process led to defining “Contexts”, in this review as the settings (e.g., education, health) or places (e.g., country, region) where programs took place. “Mechanisms” were strategies such as interventions, policies, incentives implemented to facilitate employment of youth with disabilities, and “Outcomes” were all the direct and indirect results described in the studies that related to employment. The analysis of intersectionality suffused the interpretation of how individual attributes act in the microsystems and influence the experiences of youth, the availability of programs, and expected outcomes. However limited information about the GBA+ factors limited the depth of this analysis, reducing the generalizability of our findings, and pointing to an important gap in the research literature. A critical analysis of the limited consideration for intersectionality is granted. Supplementary Table 3 provides program participants' characteristics for the included studies. Table 4 offers characteristics of the included studies including design and program objectives.

**Table 1:** Definitions of CHILD-CHII Macro-Level factors.

**CHILD-CHII Macro-Level factors**	**Definitions**
Transportation	Travel training, transportation navigation opportunities, class curriculums including transportation routes
Transportation staff training	Staff training of transportation staff and personnel considered within the program contents
Community design	Physical infrastructure of the program such as bikes routes, paths and trails
Awareness initiatives	Programs generating dialogue between youth and service providers/policy makers
Access to healthcare	Elements relating to access to healthcare and health promotion
General programs and services	Elements of internships, job placements, career counseling, job placements/training, summer employment and services
Volunteer/work	Elements related to work skills training
Education	Education at multiple levels (high-school, college/university, and/or vocational schools)
Technology	Elements relating to technology within program curriculums (e.g., computer-skills training) and/or technology used as medium for delivery of program
Web-mapping	Elements relating to the virtual linking of datasets, resources or systems
Social	Opportunities for youth to interact with other program participants, attitudes and stigmas toward persona with disabilities, opportunities for families to participate in activities
Accessibility policies	Accessibility policies/initiatives within the community

**Table 2:** Included articles organized by CHILD-CHII Macro-Level factors.

**CHILD-CHII Macro-Level factors**	**# of studies addressing**	**References**
Transportation	8	(30, 34, 40, 68, 73–76)
Transportation staff training	0	NA
Community Design	0	NA
Awareness Initiatives	0	NA
Access to healthcare	9	(28, 36, 40, 50, 51, 54, 70, 72, 75)
General programs and services	38	(26, 28, 30–32, 36–38, 40, 41, 43, 46–55, 57, 58, 60–63, 65–67, 69, 72–78)
Volunteer/work	56	All included articles
Education	25	(23, 25, 28–31, 33, 38–40, 43–47, 53, 54, 58–60, 68, 71, 75, 77)
Technology	8	(27, 29, 30, 33, 35, 40, 73, 75)
Web-mapping	1	(30)
Social	14	(30, 31, 37, 42, 56–58, 60, 64, 68, 71–73, 95)
Accessibility policies	1	(68)

**Table 3:** Main context-mechanism-outcomes structures of included studies.

**Authors, year, country, reference**	**Context**	**Mechanism**	**Outcome**
Akinola and Doabler, 2023, United States, (76)	Age, gender, race/ethnicity, social security recipient status, education level and severity of disability	Employment, academic, and disability-related elements, work placement	Education, treatment, job skills training supported employment, and social support improved employment and wages. White men had better employment, less for ethnical minorities. Transportation was not significant for employment outcomes
Almalki, 2021, United States, (32)	Mercy Medical Center, Midwest	Employment, social and personal elements, work placement	Increase in social and vocational skills and job securement
Anand and Honeycutt, 2020, United States, (47)	Disability, VR, gender, age, education, education supports, race/ethnicity, disability benefit status	Academic and employment elements	Education and academic support led to successful employment. Lower employment rates for youth with mental health support
Ashburner et al., 2018, Australia, (71)	Community-based setting, Queensland.	Academic and social elements.	Development of skills, motivation and understanding of future options, difficulties with expressing goals for some.
Balcazar et al., 2012, United States, (58)	Summer Transition Institute, Chicago, urban setting	Academic, employment, personal and social elements, work, placement	Increase in employment, wages, and education enrollment. Lower functioning participants worked less hours, were paid less. Case managers supported throughout
Barnard-Brak et al., 2023, United States, (23)	Collegiate program, University of Alabama	Academic and employment elements, work-placement	Job skills with job task analysis performance predictor of employment. Severity of impairment, not related to employment outcome
Belisle et al., 2023, ^*^United States, (48)	Alternative day-treatment program	Employment elements	Improved ability to correctly match job positions to photos
Benson et al., 2021, NR (95)	Community-based setting	Interviews	Lack of guidance during transitions, schools not catering services to child. Limited support from service centers
Berastegui et al., 2023, Spain, (39)	University	Academic elements	Moderate level of social competency development, increase in employment with time, after program completion
Black et al., 2017, United Kingdom, (68)	Shopping precinct local to the Greenside School	Independence and social elements	Development of learning opportunities, understandings of incorporating learnt material in business setting, independence, work and social skills
Burke-Miller et al. 2012, United States, (61)	Age, city study sites	Employment elements	No differentiation in likelihood of getting employed for youth with disabilities vs. older adults
Cairns et al., 2017, Australia, (70)	Illness, cognition age, gender, education, parent employment, family history, substance use, Headspace center	Disability-related elements	Education led to employment. Being female, higher parent occupation status, no recent cannabis use, and better memory led to employment and higher salaries
Carter et al., 2011, United States, (63)	Disability, age, gender, race/ethnicity, employment and independence skills, summer programs, mid-western state	Work placement	Majority attained/kept employment. Disability type not correlated with weekly earnings, but employment skills and being older were
Cmar and McDonnall, 2019, United States, (56)	Summer program, large city	Employment and social elements	Increase in self-efficacy for job searching
Cmar and McDonnall, 2021, United States, (57)	Summer program, large city	Employment and social elements	No demonstration that intervention impacted job search behavior or self-efficacy
Devine et al., 2018, United States, (33)	University office of University of Nevada, Las Vegas	Employment elements	Text comprehension levels varied amongst participants
Di Rezze et al., 2023, Canada (37)	Campus of McMaster University	Employment elements and work placement	Improvement of social skills, self-determination, understanding the workforce
Estrada-Hernandez et al., 2008, United States, (62)	Severity of disability, program, Midwestern State	Employment elements and work placement	Job placements matched participants' interests. No effect of disability severity on wages
Gold et al., 2013, United States, (78)	Age, gender, disability, education support, previous work, household income and program year race/ethnicity (multiple cities)	Employment elements and work placement	The rate of employment from program was higher than other studies of high school employment
Hanson et al., 2021, United Kingdom, (46)	Supported Internship Organization	Work placement	Improved perceived personal capacities, work satisfaction, fulfillment and self-determination
Harun et al., 2019, (45)	Secondary school graduates of education programs in Kuala Lumpur and Selangor	Interviews	Significant associations with employment, gender, family income, educational levels of mothers, parent expectations, financial support, vocational training and employment services
Honsbeger et al., 2019, Malaysia, (43)	Workplace, food truck in school parking lot	Employment elements, work placement	Improved independence and employment skills
Jonsson, 2021, Sweden, (54)	Adolescent Psychiatric unit, and ADHD unit of Northern Stockholm, Psychiatric Service, Stockholm	Social and independence elements	Levels reached beyond initial set goals, some goals did not carry through until end of program
Kaehne and Beyer, England 2013, (66)	Workplace	Work placement	Improvement of social skills and independence. Employment seen as low priority and higher education as higher. Inconsistencies in weighing funding options by all stakeholders
Kiegaldie et al., 2023, Australia, (53)	Hospital work setting, Melbourne	Interviews with students' supervisors on work placements	Increase in communication skills, reading, technology use, problem solving as well as organization, self-determination and motivation, and an increase in employment
Lee et al., 2019, ^*^Australia, (69)	Workplace in organization	Work placement	Increase in confidence, understanding of the workforce, positive feeling toward contributing to work, society and a realization of capacities
Lindsay et al., 2012, Canada, (52)	Children's hospital, Ontario	Work placement	Increase in social skills, communication and self-confidence, practical skills for employment and ability to disclose disability and ask for accommodations
Lindsay et al., 2013, Canada, (51)	Workplace, pediatric hospital, Ontario	Independence elements, work placement	Majority of youth able to disclose their disabilities within a work context and ask for required accommodations
Lombardi et al., 2017, United States, (29)	Schools, Connecticut	Employment and independence elements	Increase in employment, academic and independence knowledge
Luecking et al., 2018, United States, (28)	School setting, Maryland	Employment, social and academic elements, work placement	Increase in employment, higher obtention of IEP shorter times between processing and obtention of IEP, higher likelihood of receipt of job assistance and support, lower costs of services
Milbourn et al., 2020, Australia, (64)	Seven metropolitan Men's Sheds	Social elements	Increase in social skills, woodworking and use of tools
Mlynaryk et al., 2017, Canada, (36)	Specialized high school, Quebec	Employment elements	An active involvement of the school's staff in activities with external partners was found as facilitator to employment transitions
Muller and VanGilder, 2014, United States, (26)	Hospital, non-public school and adult service provider, Washington, D.C.	Work placement	Half of interns received employment. Increase in confidence, self-esteem, motivation and increase of understanding of work
Muthumbi, 2008, United States, (25)	Community settings in urban and suburban/rural areas, New York State	Employment and academic elements	Increase in service awareness, ability to link youth with services, employer understandings of disability needs, skill acquisition for youth and employability. Career assessments and programs range widely amongst schools
Nicholas et al., 2019, Canada, (67)	After school, workplace	Employment elements, work placement	Increase in job readiness skills, human interaction and confidence
Pebdani, 2014, United States, (55)	Workplaces in urban cities	Employment elements, work placement	Participants with previous vocational education more likely to terminate program participation prematurely
Randall et al., 2020, United States, (27)	Southeastern University	Employment and independence elements	Increase in office-related skills
Rogan et al., 2014, United States, (30)	Urban university campus, Indianapolis	Employment, academic, and independence elements	Employment rates vary year to year for participants, improvement of career preparation, independence, academics, computer, self-determination and social skills
Rumrill et al., 2016, ^*^United States, (50)	Age, gender, race/ethnicity, depression/mood disorders education level disability benefits	Disability-related, academic, social and employment elements	Those successfully employed received more services, vocational services and less time in rehabilitation. Educational and disability benefits linked with successful employment, age, gender and race/ethnicity were not
Scanlon and Doyle, 2021, Ireland, (44)	Two special schools	Academic and employment elements	Increase in confidence and certainty about planning for the future
Schall et al., 2020, United States, (77)	Four hospitals, Virginia	Academic elements and work placement	Increase in employment rate, social skills, higher self-management and technology use skills
Schillaci et al., 2021, United States, (31)	College, Massachusetts	Employment, social disability-related and academic elements, work placement	Improvement of self-initiation and volitional action autonomy, independence, self-determination
Schlegelmilch et al., 2021, United States, (72)	Multiple community/service settings, Wisconsin	Independence, social disability-related and employment elements	Increase in confidence and positive outlook for futures. Services for family key to successful employment. One participant faced barriers to employment and program because of medical condition
Shatil et al., 2023, Bangladesh, (42)	School	Academic, employment and social elements, work placement	Employment rate for disabled women and girls lower than disabled men, facing social exclusion, are more vulnerable to sexual harassment, and multiple discriminations
Skellern and Astbury, 2014, United Kingdom, (41)	College	Independence and employment elements	Increase in career preparation, independence and confidence
Strater and Elfers, 2019, United States, (60)	Kentucky distribution center	Social and employment elements, work placement	Increase in self-determination goal development, job securement, and work independence
Strickland et al., 2013, United States, (35)	Web-program, Emory university	Employment elements	Development of interview skills
Traina et al., 2022, Ireland, (40)	National University of Ireland Galway, and disability service provider	Employment and independence elements, work placement	Improvements of social, communication, independence and employment skills
Trainor et al., 2008, United States, (59)	Summer workplace, other community settings, Midwestern states	Employment elements	Curriculums not focused on youth needs; programs' inclusion criteria's non-inclusive
Versnel et al., 2008, Canada, (38)	High school in suburban Ontario Workplaces	Work placement, academic elements	Workplace had poor support, student unaware how to self-advocate
Vigna et al., 2023, Wales, (65)	Previous work, disability, school/difficulties, special needs status at school, employment preferences	Independence and employment elements, work placement	Employment rate higher for LD, and ASD+LD disabilities, than for ASD+ID. Previous work experience, and longer experience improves employment. LD more likely to obtain significant employment vs. ASD and ID. No difference with age and gender
Wehman et al., 2014, United States, (74)	Suburban hospital, Richmond, Virginia	Independence elements, work placement	Increase in work independence and employment
Wehman et al., 2012, United States, (49)	Suburban hospital, Virginia.	Employment elements, work placement	Increase in job securement
Wehman et al., 2017, (73)	Hospital.	Employment, social and independence elements, work placement	Increase in hours worked and wages, decrease in support needed
Wehman et al., 2019, United States, (75)	Four hospitals, Virginia	Employment, independence, disability-related academic and social elements, work placement	Majority obtained employment, higher wages and hours worked overtime
Wilson et al. 2017, United States, (34)	Workplace, Delgado Community College, urban campus	Employment, academic and independence elements	No reported results, article overviews a program

^*^When program country NR, first author's country of affiliation given.

Program elements were grouped into categories, for full program details, see Supplementary Table 2.

Employment elements: e.g., employment skills training.

Work placements: e.g., internships, apprenticeship.

Academic elements: e.g., classroom training, college support.

Independence elements: e.g., transportation training, financial coaching.

Disability-related elements: e.g., diagnosis services, early intervention service.

Social elements: e.g., social skills training courses, social gatherings for youth.

**Table 4:** Included articles' study characteristics.

**Authors, year, country, reference**	**Design**	**Objective**
Akinola and Doabler, 2023, United States, (76).	NR, secondary data analysis	Research whether demographic factors play a role in successful employment
Almalki, 2021, United States, (32)	NR, phenomenological approach	Improve employability skills, research perceptions of employees and coworkers on project practices
Anand and Honeycutt, 2020, United States, (47)	Longitudinal study	Explore long-term employment outcomes for youth with and without mental health conditions
Ashburner et al., 2018, Australia, (71)	Qualitative program evaluation	Examine program's impact on participation, emotional state, project skills, and knowledge of transition options
Balcazar et al., 2012, United States, (58)	Pre-post control group	Develop and field test transition skills management program
Barnard-Brak et al., 2023, United States, (23)	NR, predictive correlational study	Examine components of program that predict employment
Belisle et al., 2023, ^*^United States, (48)	Non-concurrent multiple baseline across employment settings	Evaluate a Equivalence-based instruction work-support program
Benson et al., 2021, NR (95)	Phenomenological	Understand parent perspectives on transition processes
Berastegui et al., 2023, Spain, (39)	Longitudinal study	Link career trajectories to competencies
Black et al., 2017, United Kingdom, (68)	NR, case study	Discuss education paths, describe project
Burke-Miller et al. 2012, United States, (61)	Multisite randomized controlled trial	Examine supported employment for employment outcomes
Cairns et al., 2017, Australia, (70)	Cross-sectional, prospective investigation	Identify links between education and employment before and after looking at demographical and clinical factors
Carter et al., 2011, United States, (63)	NR, longitudinal study	Gather information on employment and summer activities
Cmar and McDonnall, 2019, United States, (56)	Quasi-experimental	Evaluate program' effectiveness in increased job search knowledge, behavior and self-efficacy
Cmar and McDonnall, 2021, United States, (57)	Longitudinal study, two-group, quasi-experimental repeated-measures	Examine effectiveness of training over time, based on job-search behavior, knowledge and employment
Devine et al., 2018, United States, (33)	Multiple probe across participants	Evaluate effects of reading out loud of handbook, with an instruction-based literacy treatment package for text comprehension
Di Rezze et al., 2023, Canada, (37)	Pilot study	Co-design program and evaluate how it helps support and develop job skills
Estrada-Hernandez et al., 2008, United States, (62)	NR, observational study	Explore disability severity in relation to employment outcomes
Gold et al., 2013, United States, (78)	NR, cross-sectional	Examine whether program participants obtain jobs and if demographic factors predict program placement
Hanson et al., 2021, United Kingdom, (46)	Case-study	Research social exclusion experiences for youth with learning disabilities
Harun et al., 2019, (45)	Cross-sectional	Evaluate employment experiences, job retention and other factors
Honsbeger et al., 2019, Malaysia, (43)	Multiple probe	Explore efficacy of program in increasing and maintaining work skills
Jonsson, 2021, Sweden, (54)	Open feasibility study without control group	Evaluate program feasibility
Kaehne and Beyer, England 2013, (66)	Pilot study	Research perceptions of parents and employers about supported employment
Kiegaldie et al., 2023, Australia, (53)	Pilot study	Research how program supported youth for employment, and how it compared to other work models
Lee et al., 2019, ^*^Australia, (69)	Grounded theory	Explore factors relating to employment and experiences of participants
Lindsay et al., 2012, Canada, (52)	Descriptive qualitative methodology	Research skills acquired after program completion, and perspectives on what can be changed to improve it
Lindsay et al., 2013, Canada, (51)	Descriptive qualitative methodology	Research if youth with disabilities disclose their conditions and what accommodations they request in workplace
Lombardi et al., 2017, United States, (29)	Quasi-experimental	Examine effectiveness of program
Luecking et al., 2018, United States, (28)	Non-experimental impact analysis	Examine effects of intervention
Milbourn et al., 2020, Australia, (64)	Qualitative, pre-post study	Examine occupational experiences of participants
Mlynaryk et al., 2017, Canada, (36)	Qualitative descriptive design	Understand perspectives on what leads to successful transition
Muller and VanGilder, 2014, United States, (26)	Qualitative and quantitative-mixed-methods evaluation	Research link between program and job readiness
Muthumbi, 2008, United States, (25)	NR, descriptive overview of program	Demonstrate successful services for the employment of youth with a variety of disabilities
Nicholas et al., 2019, Canada, (67)	Secondary review of anonymized program evaluation outcomes	Review pre-employment program
Pebdani, 2014, United States, (55)	NR, non-experimental impact analysis	Explore what leads to early termination in works programs, and if personal factors contribute to this
Randall et al., 2020, United States, (27)	Multiple baseline design	Examine effects of Task Analysis app for office tasks
Rogan et al., 2014, United States, (30)	NR, case-study	Describe program
Rumrill et al., 2016, ^*^United States, (50)	Purposeful selection of multivariate logistic regression	Research relationships between demographics and a vocational rehabilitation program
Scanlon and Doyle, 2021, Ireland, (44)	NR, descriptive study	Report on student perspectives from program, and how it impacted decision making skills
Schall et al., 2020, United States, (77)	Randomized controlled trial	Describe findings and employment outcomes of internship program
Schillaci et al., 2021, United States, (31)	Quasi-experimental research design	Research self-determination in program participants vs. participants in a transition program not in a college setting
Schlegelmilch et al., 2021, United States, (72)	Qualitative multiple case study	Explore participant's perspectives of participation in intervention
Shatil et al., 2023, Bangladesh, (42)	Qualitative study	Examine effects of social inclusion and economic exclusion
Skellern and Astbury, 2014, United Kingdom, (41)	Qualitative study	Research employment experiences from participants in program, examine employment obtention strategies
Strater and Elfers, 2019, United States, (60)	Sequential exploratory	Examine how program affects self-determination in participants
Strickland et al., 2013, United States, (35)	Randomized controlled trial	Research program effectiveness in teaching interview skills
Traina et al., 2022, Ireland, (40)	Pilot study	Evaluate effectiveness of program in terms of inclusion and employment skills
Trainor et al., 2008, United States, (59)	Qualitative interview study	Research perspectives of special educators in support of employment and community activities
Versnel et al., 2008, Canada, (38)	Case studies	Describe experiences of participants
Vigna et al., 2023, Wales, (65)	Observational study	Research determinants of employment obtention
Wehman et al., 2014, United States, (74)	Randomized clinical design	Examine effectives of employment from project vs. individualized education programs
Wehman et al., 2012, United States, (49)	Case study	Describe project
Wehman et al., 2017, (73)	Randomized controlled trial	Design and implement program
Wehman et al., 2019, United States, (75)	Randomized clinical trial	Research employment training and outcomes
Wilson et al. 2017, United States, (34)	NR	Describe program

^*^When program country NR, first author's country of affiliation given.

Bellow we describe a summary analysis of contexts, mechanisms, and outcomes, and our iterative programme theory development, anchored on the CHILD-CHII and GBA+ frameworks.

### 3.1 Context, mechanisms (CHILD-CHII Macro-Level factors) and outcomes: an explanatory synthesis

#### 3.1.1 Context

Programs were held primarily in educational settings (*n* = 21), in the United States (*n* = 12) (23, 25–35) Canada (*n* = 3) (36–38), Spain (*n* = 1) (39), and the United Kingdom (*n* = 2) (40, 41) with only two reported rural areas outside of North America or Europe (*n* = 2), in Bangladesh (42) and Malaysia (43).

Educational settings included colleges and (23, 27, 30–35, 37, 39–41) a high school (38) a specialized high school (36, 44), a non-public school (26), an educational agency (25), a specialized education setting (45) and other non-described school settings (28, 29). Programs were also held in hospital settings (*n* = 10) including the United States (*n* = 6) (26, 46–50) Canada (*n* = 2) (51, 52) Australia (*n* = 1) (53) and Sweden (*n* = 1) (54).

Community-based settings also included workplaces, in the United States (*n* = 11) in urban (55–58) suburban (25) and unspecified (59–63) areas in the country, and urban areas in Australia (*n* = 1) (64). Other work settings were in the United Kingdom (*n* = 3) (40, 65, 66) Australia (*n* = 3), Canada (*n* = 2) (38, 67) and rural Malaysia (*n* = 1) (43).

Examples of community-based settings were summer training programs (58), a local work consortium and independent living center (25), a shopping precinct close to a school (68), a food truck in a school parking lot in (43), an Information and Communication Technology-related organization (69), a youth health center (70), an alternative day-treatment program (48), a disability service provider (40), and community-based settings (46, 47, 71, 72).

The contexts where these programs take place present a clear predominance, and likely selection bias, of macrosystems that translate North American, Western, urban values, economic and legislative systems. Such contexts describe a community environment where the micro context of family and their youth must navigate a set pathway in formal structures of care—with strong mediation of both educational and health systems. In these contexts, there are limited naturally occurring opportunities for interactions with neighbors, peers without disabilities, ethnic or religious groups—spaces where inclusivity, accessibility and related mechanisms associated with employment occur. An analysis of these limitations is presented later in this manuscript.

#### 3.1.2 Mechanisms

##### 3.1.2.1 Transportation

Transportation was included in programs' curriculums in the form of travel and familiarization/navigation of transportation routes to and from program location (30, 40) road safety, reading maps (40), and bus schedules (73) group exercises on public transportation, learning how to travel independently between different environments including the work environment and other life spaces (34, 40, 68, 74). Only one study addressed the microsystems of families, inviting with parents to discuss employment and transportation relationships (75). One program assessed the mediating impact of familiarity with public transit services and employment outcomes, with no conclusive mechanisms connecting transportation and earnings, but an indication that ability to retain employment could be related to ability to use transportation independently (76). Beyond a mediating factor, transportation was also seen as an outcome, with a study reporting an increase in ability to navigate the city after program completion (30).

Although we theorize that transportation is a mediating mechanism contributing to employment outcomes on the exosystem of western, urban contexts, numerous studies did address transportation (40, 73–75). We also note that public vs. private transportation are very distinct variables, and may imply specific resources and various levels of micro-meso-exo systems interactions depending on type of disability, urban contexts, possibility for active transportation, existence of adapted public transit systems, financial means and supports for individual transportation and the rules, regulations and affordability of each of these systems—none of which were explored in the studies identified.

##### 3.1.2.2 Access to healthcare

Access to healthcare and other health considerations emerged within program curriculums, mostly related to the offer of rehabilitation services, social work support (36), teaching topics of healthy living or healthy lifestyle group discussions (40, 54) and tackling topics related to substance, mental health and general health issues (70), including health promotion training (72), setting health goals (54) and working with staff and employers and healthcare providers to help set up strategies for self-care (51), and medical (50) and rehabilitation services (28). Once more, the project that addressed parental training included discussions with parents on how employment of their youth could impact public health benefits, being the one study that attempted to extrapolate their programs from micro to exosystems considerations (75).

Including health-related factors in the programming did not seem to connect to specific employment outcomes, but rather informed health-related outcomes such as setting goals for healthy living, going to the gym more regularly (54). However, addressing the domain of “health” within the microsystems of individual health promotion, may be an indirect mechanism related to employment outcomes when individuals have the opportunity, within the mesosystem of the work environment, to address facts such as fatigue from long work-days, managing pain, organizing personal time and accessing the needed supports to maintain their health in the work environment (51). The underlying mechanism allowing for these health accommodations might relate an intentional disclosure of disabilities within the work context, direct access to health services, and the possibility to request the necessary accommodations (51). On the contrary, when youth experienced abrupt interruption of rehabilitation and social work supports upon graduation of employment programs, they experienced limitations in the ability to manage their health (36).

Only one study reported health mechanisms acting in the exosystem through coordination between mental health services, disability services and rehabilitation providers facilitated mesosystem outcomes such as expanding eligibility criteria for employment services, and reducing the cost of services for participants (28). This finding confirms our theory that coordination of systems of care and community configurations through sectors (i.e., health, education, employment, social work residing in siloed vs. confluent structures) is a major player in employment outcomes. Considerations of how intersectionality plays a role in the functioning of such systems was not addressed in the studies, hindering our ability to testing of how the additional structures involved in exosystems dealing with GBA+ factors (e.g., systems of immigration, indigenous health authorities) may represent additional barriers or facilitator mechanisms to the employment pathways.

##### 3.1.2.3 Education

Education was included in program curriculums including *skills training* such as reading comprehension (29), teaching text comprehension of an adapted employee handbook (33), group workshops on education themes (54). Other skills covered were academic and classroom training (30, 42), communication, social skills and independence (68). Education was also incorporated into employment programs that facilitated formal continuing education such as visiting tertiary educational institutions (71), school placements (38, 46) and opportunities to earn degrees (34, 39) coordination with school staff to supplement regular education services (28) and provide vocational support and job readiness and counseling (25, 36, 44, 60). Additional supports included access to special education teachers alongside adult services (75), accommodations from the university's disability office (31), education coaches (31), and support by special education staff (58, 77) through program curriculum.

Studies that focused on education-related mechanism reported an increase in employment rates ranging from 42% (31) to 75% (28, 30, 58, 60, 75, 77). Other documented positive outcomes included a higher increase in employment after program completion (39), a moderate level of social competency development (39). Programs also had positive effects on soft skills like social participation (58), social skills (77), motivation (71), emotional well-being (58), confidence (44), improved perceived personal capacities (46). Additional improvements included increased self-initiation and volitional action autonomy (31), improvement in work satisfaction and fulfillment (46), independence (31, 43), self-determination (31, 46, 60), self-esteem (46), an increase in self-awareness to challenges (60), higher self-management (77), goal attainment and higher than initially set one of the goals (54), certainty about planning for the future (44), and a development of understanding of future options (71).

Mechanisms operating in the microsystems of developing individual skills such as reading, writing strategies, test taking, technology use, cooking and retail—related to outcomes also at the microsystems of self-efficacy, with limited report on mesosystem or direct employment of the acquired skills (29, 58, 68, 77).

One study found that participating in job skills training, paired with job-specific task analysis was a predictor of employment when compared to youth who were unemployed or worked in a sheltered workshop environment (23). Meso-exosystems were impacted through employer's increased awareness, ability to link youth with services, and understandings of disability needs. Active involvement of the school's staff in activities with external partners (36) was a mechanism leading to easier school to work transitions for youth.

Negative outcomes emerged when education mechanisms were not in place. For instance, if employers did not have adequate education about their employee's disability needs the workplace did not effectively support the students in doing their jobs, or if the youth did not have adequate education to be prepared for the work placement. There were also reports of misaligned expectations between supervisors and youth, as well as limited knowledge among youth about their rights and how to request needed accommodations. This often led to unmet goals and tensions between employers and students (38, 54, 71). Microsystems represented by individual variability in educational attainment influenced the work-related outcomes (33).

##### 3.1.2.4 Technology

Use of technology was a mediator mechanism to teach and deliver specific tasks, but was not a primary mechanism presented in the studies. For instance, programs provided participants with step-by-step pictures, video or audio of instructions and tasks (27, 35, 40, 73), deliver comprehension exercises (33) and teach online career/vocational readiness and exploration and IT literacy (29). One program used quizzes, and scenarios to support youth in learning interview skills (35), facilitating use of on-campus computers to update journals to increase familiarity with technology used by students (30). Assistive technology such as specific software and hardware, was also a mechanism to support youth in their job-related tasks and to facilitate the preparation of a presentation for non-verbal participants (40, 58, 75).

Outcomes mediated by technology-related mechanisms were reported most at the microsystems through participants' improvement in office-related skills such as photocopying or scanning (27), computer skills (29, 30). Potential mesosystems outcomes were reported through resume writing skills (29) and text comprehension skills (33), which may be transferable to other environments and tasks, though these outcomes at the mesosystems level were not tested or reported. Access to appropriate assistive technology obtained in rehabilitation was associated with successful employment, supporting the hypothesis of some level of interaction across micro and mesosystems (50).

##### 3.1.2.5 Social

Social aspects were included in studies through different mechanisms: giving youth opportunities to socialize (31, 58, 60, 68, 71), or group activities to build social skills and supports (56). Opportunities to develop social skills were introduced through different mechanisms: support individual meetings with staff (31), hiring students for peer-support assistance for note taking, school-work, making friends, and event attendance (30) mentorship (31, 64, 73) and having access to tutors and coaches (31). Social aspects were also included in training opportunities, in group activities discussing self-efficacy and social support (57), self-determination (60), self-advocacy (37, 72) family advocacy (72), employer-employee workplace relationships (42), professionalism and expectations (37), recruiting mentors or helpers (58), dealing with social issues and stigmas, workplace activities (42), and courses to develop communication, social and independence skills (32, 68).

Outcomes reported had higher potential for cross-systems interactions and demonstrated a pathway of interaction across micro and mesosystems through improved individual skills being transferred to other environments: social skills (37, 58, 64, 68), increased self-determination (30, 31, 37, 60) well-being (58), motivation (71) and confidence (72), understanding the workforce (37), independence (30), self-efficacy for job searching (56), development of social goals by the participants (60), reduced support intensity needed for participants throughout the program (73). Hard skills were also reported as outcomes in programs that emphasized social-related outcomes, such as woodworking and use of tools (64) and other specific task-related skills development (30, 46, 68, 71). Increased employability was another outcome reported in some of these studies, perceived as an outcome of the social skills training (30, 58).

Null outcomes were also reported, with a lack of transferability of social skills to employment outcomes. Youth participating in a program working on social skills did not present changes in job search behavior or improvement in self-efficacy (57), prompting the need to explore the specific mechanisms involved in the transferability process, beyond cross-sectional or qualitative measurement.

##### 3.1.2.6 Web-mapping

Only one study described the use of web-mapping in supporting employment programs. In this study, a web-based resource was used to link available programs and networks to circulate information on post-secondary education for youth with intellectual disabilities and collect data on outcomes for social inclusion, achievements within projects from the resources in the network, and career outcomes (30).

This research reported an improvement in work skills, employment readiness and career preparation, self-determination and social skills of participants, as reported by class instructors at the end of the university semester (30).

##### 3.1.2.7 Accessibility policies

Only one program included considerations of exosystem mechanisms though the existence of accessibility policy. The study analyzed the school's policy documentation to understand outcomes of the first year of a vocational support program for youth with learning disabilities (68). Mechanisms identified included integrating broader life skills such as self-development and independence into curriculums in addition to specific vocational training (68). One limitation discussed by authors was the possibility that this might reinforce pre-conceived notions that youth with learning disabilities should focus on practical skills rather than life skills, indicating a relevant interaction of the macrosystem, with values and understanding of disabilities functioning as a mechanism into the employment programs. However little was established into how this interaction actually influenced the employment outcomes (68).

##### 3.1.2.8 General programs and services

General programs and services related to other opportunities related to employment, that were not specific about one skill or one career path. These opportunities provided clearer mechanisms for youth to experiment the interactions beyond the mesosystem of the protected programs to the exosystem of “real” job opportunities. Activities favoring these mechanism included psychological counseling (50, 65, 76), career guidance and preparation (30, 43, 69, 78), coached services/job opportunities (31), internships (46) and paid work (37), staff assistance within job search, interviews, and placements (57, 78) work placements (38, 40, 50–52, 55, 65, 66, 76), job shadowing (62, 78), integrated employment (28) and supported employment (61), summer employment (63), paid apprenticeship (34) and internships (26, 30–32, 49, 53, 60, 69, 73–75, 77), job skills (32, 49, 67), vocational (50, 62, 76) and on the job-training (62), employment retention services (50), financial support (e.g., tuition/supply payment) (47), and vocational assessment (65).

Other mechanisms acting in the interaction of meso and exosystems included working on life skills (41) and independent living (32, 41), development of personal plans based on personal interest to help in career guidance (62), financial coaching and work benefits (72), household management/housing leisure workshops (65). Important mechanisms to facilitate the movement from micro to exosystem included teaching youth to set goals and plan actions in life (58), soft skills training such as interpersonal relationships and communication (72), and identification of personal strengths (58). To facilitate youth action into the exosystem, some programs included teaching about disability services (31), benefit planning and determining career beneficiary aid (36, 65), and seeking assessment and diagnosis that can support accessing benefits (50).

The mechanisms acting on the interaction of systems were perceived as having a large impact on the microsystems, particularly on the way youth perceived themselves and their immediate environment of families and peers perceived them. Few programs tested specific mechanisms leading to target outcomes, for instance, one study demonstrated that participating in a program where participants were tasked with matching photos of employment personnel to their job descriptions led to an greater understanding of the workforce, as shown by an improved ability to correctly match job positions to photos (48). Other general outcomes reported in relation to non-work specific program content included social inclusion, (46), self-esteem (26, 46), social (32, 52, 66, 67) and vocational skills (32, 67), career preparation (30, 41), independence, confidence (26, 41, 52, 67, 69), motivation (26) understanding of the workforce (26, 48, 55), positive feeling toward contributing to work, society, and an realization of capacities and perspectives for the future (69, 72). Direct employment-related outcomes included increase in practical skills for employment (interview, office skills, resume writing, organizing transportation, ability to disclose disability and ask for accommodations (52)), increase in job searching self-efficacy (57), improvements of work independence (60) and job securement (50, 63, 65, 78), and obtaining job placements that matched their interest (62). Outcomes on the mesosystem included lower cost of services when youth were matched with job assistance and support (28), indicating a potential for positive outcomes at the exosystem when mechanisms include intentional micro and mesosystems interactions.

Negative outcomes included youth who had previous vocational education being more likely to leave a program prematurely (55)—however little exploration of the mechanisms leading to these outcomes were noted. Little consideration was given to the micro and mesosystems interactions in most studies. Programs that includeād families reported that parental perception of priorities influencing the youth employment outcomes. Depending on the age of youth, parents may want to focus energy on transition to higher education rather than a job (66), revealing how the microsystem of families of youth with disabilities are a strong intervening factor at this transition age.

#### 3.1.3 Explanatory CMO configurations

Our analysis identified several recurring CMO configurations described above. We draw attention to two particular configurations that transit in opposite directions under an ecological theory perspective. One configuration, supporting segregated contexts, with mechanisms that stay within micro and mesosystems at most, and involve the acquisition of specific skills and sheltered job-related opportunities, also opposes a human rights theory, where the notion of “positive rights”—the existence of fully integrated, inclusive contexts affirming the right of every human to be where they chose to be—supports the existence of inclusive, not segregated systems. The other CMO configuration assumes some level of real-world contexts, or with a larger interaction of meso and exosystems. In this second primary CMO, human rights principles are enabled through policies and inclusion by design, though studies only marginally considered factors in the macrosystem as part of their intentional mechanisms. The outcomes reported in these CMO configurations may consider how youth with disabilities could bring to the exosystems when they acquire skills, conquer positions and change work place culture and policies to integrate their needs and capitalize their potential—however the impact of the macrosystems of employment, including values such as productivity, sustained economic impact, and competitiveness are not deeply considered.

##### 3.1.3.1 Skills training in protected environments

- Context: Highly structured and protected settings, such as specialized schools (36, 44), hospitals (46, 51), and universities with strong disability support services (30, 31, 34).
- Mechanism: The primary mechanism activated in these contexts is the development of self-efficacy and individual skills that can potentially be transferred into employment opportunities. Through structured skills training (e.g., CV writing, interview practice, technical skills), youth build confidence in their abilities in a low-stakes environment where mistakes are framed as learning opportunities. The expectation is that these skills will be relevant and transferred to the employment contexts.
- Outcome: The primary outcomes measured were at the micro level through participants' increase in job-readiness skills, self-determination, and confidence (44, 46). While many studies report subsequent employment (28, 60), these outcomes are often short-term or in supported settings. Transfer of these outcomes to other environments were rarely measured and cannot be confirmed.

##### 3.1.3.2 Real-world exposure with scaffolding

- Context: Community-based work placements, internships, and summer employment programs (58, 63, 74). These contexts involve real-world work environments, but include program elements that support the interactions between micro, meso, and exosystems through individual-level supports job coaches, trained supervisors, and program and systems level mechanisms such as partnerships between the employer and a support agency.
- Mechanism: The key mechanism here is the accrual of applied interpersonal skills and awareness raising in the workplace. By interacting with co-workers and supervisors, youth learn tacit workplace norms, build professional networks, and gain valuable experience that signals their employability to future employers. The presence of a job coach or supportive supervisor are also mechanisms intending to transform the workplace culture, not only the potential worker.
- Outcome: Outcomes include the development of soft skills (e.g., communication, teamwork) (67), an increased understanding of the workforce (55), and often, direct job offers from the placement site (26). This configuration appears more effective at leading to direct employment than skills training alone, suggesting that the combination of practical experience and social support is a powerful driver of success.

### 3.2 Negative, null, and inconsistent findings

A critical analysis of the evidence reveals that program success is not universal. Several studies reported negative, null, or inconsistent outcomes, or did not measure or structure outcomes beyond the micro and mesosystems, limiting a comprehension of the outcomes at the exo and macrosystems. Examples of such configurations showing negative or null findings include:

The “Skills Cliff”: A recurring pattern was the failure of skills learned in protected settings to transfer to real-world environments. For example, Versnel et al. (38) described how youth who had received training were still unable to advocate for themselves or navigate workplace challenges, leading to misaligned expectations and tension with employers. This suggests that skills training (Mechanism) in the absence of real-world practice and support (Context) is often insufficient to produce sustained employment (Outcome).

Several studies highlighted failures at the meso-system level, confirming the theory that these systems interact and indicating that when interaction fails, developmental outcomes may be limited. Mlynaryk et al. (36) reported an “abrupt interruption” of rehabilitation and social work supports upon graduation, creating a service cliff that jeopardized the transition to employment. This points to a failure of coordination between educational, health, and employment systems, where a lack of continuity in support (Context) undermines the individual gains made by youth (Outcome).

Negative outcomes also arose from a mismatch between the individual and the work environment. Lindsay et al. (51) noted challenges such as fatigue and pain management, indicating that even with individual willingness, the physical or structural realities of the workplace (Context) can create important barriers. Similarly, the exclusion of youth with more significant support needs from some programs (59) demonstrates a systemic failure to provide appropriately tailored interventions.

The review also identified findings that challenge simple causal assumptions. For instance, Rumrill et al. (50) found that transportation was not a significant predictor of employment outcomes in their sample, contrary to the theoretical assumption that transportation is a macro factor (CHILD-CHII) and a potential mediator in the exosystem interacting with employment. Similarly, while some studies found disability severity to be a barrier (58), others reported no significant relationship between impairment level and employment success (23). These inconsistencies suggest that the influence of such factors is highly context-dependent and mediated by other variables, such as the availability of alternative supports (e.g., remote work options, specialized job coaching) that were not always measured in the included studies.

### 3.3 Critical analysis of the evidence base

It is crucial to interpret the findings of this review in light of the significant concentration of the evidence base in specific contexts. The predominance of studies from urban, North American settings (*n* = 12 in the US, *n* = 3 in Canada within educational settings alone) and within formal educational and health systems, indicated a limitation of the generalizability of these CMO configurations that might work differently in various other potential contexts and macro and exosystems. The time when this review is conducted also speaks to the chronosystems in different ways. First, there were few technology-mediated interventions mentioned, which limits the timing and relevance of this review, as reality quickly evolves toward more technology-mediated mechanisms and outcomes.

The focus on high-income, Western countries means that the identified mechanisms and outcomes are likely shaped by specific policy environments, social safety nets, and cultural norms regarding disability and work. The successful mechanisms identified may not be transferable to low- or middle-income countries with different economic structures, informal labor markets, and social support systems. The near absence of evidence from rural settings also limits our understanding of how geographic isolation and lack of infrastructure impact employment pathways.

The concentration of research in educational and health settings means that our understanding of employment transitions is heavily filtered through a service-provision lens. This may overemphasize formal programs and interventions while underrepresenting more organic, community-based pathways to employment that occur outside of institutional settings. It potentially biases the findings toward individual-level skill development, which is the natural focus of such institutions, at the expense of understanding broader social and economic drivers of employment.

This skewed evidence base means that our refined program theory is strongest in explaining transitions within these specific contexts and weakest in explaining pathways in other global regions or less formal community settings. The conclusions drawn in this review must be moderated by this reality, and there is a clear and urgent need for research that examines youth employment transitions in a wider range of geographic, economic, and cultural contexts.

### 3.4 Considerations of employment and intersectionality

An intersectionality analysis showed employment rates for disabled women and girls was lower than disabled men (42, 45, 78). Woman and girls also face more severe social exclusion and are more vulnerable to sexual harassment, and to multiple discriminations (42). One study found significant associations between employment and gender, family income, the educational levels of mothers, parent expectations, financial support, vocational training and employment services (45). However, one study measuring neurocognitive, demographic and clinical factors found that being female was associated with better employment outcomes and global functioning (70).

Results were also reported for youth with more than one disability or mental health conditions in association with a developmental disability. One study reported that youth participating in vocational rehabilitation service who had a mental health condition had lower employment rates than youth with other disabilities (64). Another study showed that autistic youth who have an intellectual disability had lower employment rates comparison to youth who have an intellectual disability but were not autistic (65). One study reported that for one participant, their medical condition (physical disability, deafness, blindness and autism) was a barrier to accessing employment and the services offered by the program, although enrollment in the program made them want to work, changing their perspectives (72).

Results were also reported on disability severity. Lower functioning participants worked less hours and were paid less while receiving equal or greater support (58). In some cases, program inclusion criteria excluded participants based on severity, for example youth with less severe disabilities who did not meet eligibility requirements, or those with low-incidence disabilities whose needs were unmet because curricula focused only on functional or academic skills (59). In contrast one study found that the severity of participant's impairments, specifically in the participants intellectual and adaptive behaviors was not found to be related to employment outcomes (23).

One study that considered age and employment outcomes for factors such as education, found that for youth in comparison to older adults, (31 years and up), there were no differentiation in the likelihood of getting employed (61). Other factors associated with better employment outcomes (70, 76) and higher salaries (70) included: having a specific diagnosis, access to treatment services (76), having a higher parental occupation status, no recent use of cannabis, a better memory, complete secondary school education (70), postsecondary education (47) and access guidance and counseling (76).

### 3.5 Theory building and testing

Building on the Bronfenbrenner's Ecological Systems Theory, we can consider that the transition from school to work is a key developmental milestone that most intensely tests the interdependency of the five systems. It also represents a hallmark of the chronosystem when time typically means the individual moves from childhood to adulthood. In most western societies, employment is a pre-requisite to integrate into society as an autonomous individual. It marks the depart from microsystems that are highly dependent on families and adults in immediate spheres of care to a microsystem of work where most variables are unknown to the individuals who followed the child closely until that point. The presence of an exo and macro systems that are supportive and inclusive is fundamental to create a microsystem of work environments that are welcoming and accommodating enough to allow the individual to transition and succeed in these new life roles. The evidence in this review points to a fragmented understanding of this complexity. The majority of studies identified happen in protected educational or health/rehabilitation **contexts** with few **mechanisms** addressing the meso system and the interconnection between the family, the known contexts of specialized education and rehabilitation and real work environments. Most mechanisms identified focus at the individual level, namely skills building and capacity training. Even the mechanisms that tap into the macro and meso systems addressing aspects such as transportation, use and access of health and community services, and communication with families and employers, do so from the viewpoint of the individual. They rarely include mechanisms aimed at changing or informing broader systems, governments, norms, or economies. The **outcomes** that are associated with these mechanisms also focus on changes at the individual level, with little or no measurement or description of outcomes at the meso, macro, and exo systems.

**Figure 2:**
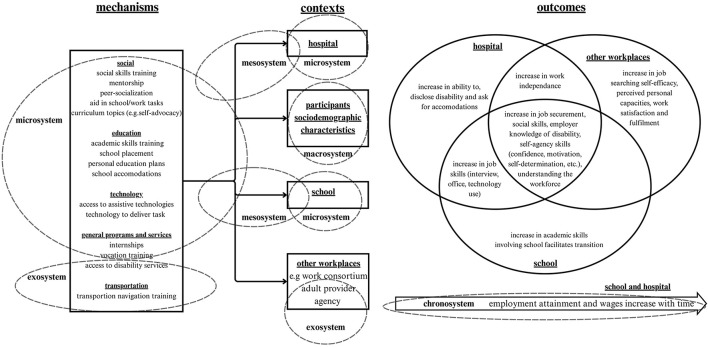
Context-mechanism-outcome relationships across Bronfenbrenner's ecological systems.

Figure 2 summarizes the relationships found across contexts, mechanisms and outcomes with Bronfenbrenner's Ecological Systems Theory.

## 4 Discussion

### 4.1 Summary of findings and refined program theory

This realist review analyzes the community factors included in school-to-work transition programs for youth with all types of disabilities. Our findings reveal that the majority of transition programs contain curriculum elements from different contexts (school, health and rehabilitation, community), often including mechanisms that focus on: (a) social interaction: opportunities for participants to engage with others in social environments (30, 31, 58, 60, 68, 71) or mentorship (31, 64, 73), (b) skills building: job-specific abilities (29, 33, 35, 36, 50, 62, 76), job-related soft skills (37, 40, 57, 60, 68, 72), self-care skills (40, 51, 54, 72), and (c) navigation: learning how to use transportation (30, 34, 40, 68, 73, 74). These program curriculum elements were found to be most prominently belonging to Bronfenbrenner's microsystem, as these curriculum elements pertain to a youth's own skills building and immediate surroundings. The outcomes targeted focus on readiness to work, employability, employment retention, and overall quality of life and well being.

Our analysis, which began with an initial program theory grounded in Ecological and Intersectionality theories, was refined through a critical synthesis of the evidence. The findings reveal that the majority of existing programs operate primarily at the microsystem level, focusing on individual skills-building and readiness for work.

Our refined, middle-range program theory can be articulated as follows: While individual-level mechanisms such as skills training are necessary components of employment support, they are insufficient to generate equitable and sustained employment outcomes. Their effectiveness is highly contingent on the presence of supportive and aligned factors at the meso and macro-levels. Successful transitions occur when individual capacity-building (microsystem) is actively bridged to real-world opportunities through scaffolding mechanisms (mesosystem), such as job coaching and employer partnerships. However, the ultimate success and scalability of these programs are constrained by broader structural and policy contexts (macrosystem), and our understanding of outcomes is limited on the inequitably application of these mechanisms across youth with different intersectional identities.

This refined theory highlights a significant gap between the comprehensive, multi-level approach suggested by our initial theory and the predominantly individualistic focus of the interventions described in the current evidence base.

### 4.2 Focus on individual-level

A major finding of this review is the overwhelming focus of existing research on individual-level mechanisms. The most frequently identified program components (e.g. skills training, job preparedness, and mentorship) are all aimed at changing the individual youth. While important, this focus inadvertently places the onus of adaptation on the person with a disability, rather than on the systems and environments that create barriers. Our analysis shows that the evidence base is largely silent on interventions aimed at tackling structural, political, and macro-level influences.

This individual-level bias is likely a product of the institutional contexts in which most research is conducted (i.e., education and healthcare), which are inherently focused on individual service provision. However, it results in a critical blind spot. The review uncovered evidence of programs failing not because of deficits in the youth, but because of systemic failures, such as the abrupt cessation of supports (36) or workplaces that were unaccommodating (38). These findings support the argument that without addressing the broader systemic determinants of employment such as inflexible workplace structures, discriminatory hiring practices, inadequate social protection policies, and inaccessible transportation, the impact of individual-level interventions will always be limited. The conclusions of this review must therefore be moderated: the pathways to employment described are predominantly pathways of individual adaptation, not systemic change.

### 4.3 Re-examining intersectionality: beyond demographics

While our review initially sought to apply an intersectional lens, the primary literature itself offered limited opportunities for a deep intersectional analysis. The majority of included studies limit their analysis to reporting demographic data, with few examining how the interlocking systems of power associated with race, gender, class, and disability status shape experiences and outcomes.

For example, the finding that young women with disabilities face lower employment rates and greater social exclusion (42) is a critical starting point, but the reviewed studies do not provide the necessary data to explain why this is the case. The mechanisms through which gender and disability intersect to produce this outcome such as gendered expectations about work, increased caring responsibilities, or heightened vulnerability to workplace harassment are not explored. Consequently, the validity of the conclusions from the primary studies is limited, as they present an incomplete picture that implicitly centers the experience of the most privileged within the disabled community (e.g., white, male youth with access to services).

### 4.4 Strengths, limitations and future directions

This review provides an in-depth account of the **context, mechanisms**, and **outcomes** surrounding employment programs for youth with disabilities. This review was inclusive of all disabilities and contexts of practice, adopting a realist approach. It allows for theoretical understanding of what works and for whom in terms of school-to-work employment programs for youth with disabilities. Across the literature, information on program settings varied, limiting the understanding of meso and macro elements of contexts relating to specific mechanisms and outcomes. Certain studies provided very detailed accounts of the program areas (urban setting, suburban location, etc.) while other provided only a broader geographical location (country, province, etc.). The lack of information for meso context such as neighborhoods and associated demographical data, or macro, such as public policies and government structures hinders the ability to gain a more in-depth understanding of the “where” these mechanisms may work, which is something that should be explored in future studies.

Certain limitations to the review include a narrowing in the scope to focus on programs, due to the large number of articles, rather than all literature discussing youth employment pathways. We also limited the review to peer-reviewed research articles, limiting the inclusion of practices that are happening outside of research contexts. Information published in gray literature, such as government reports, or other non-peer-reviewed mediums was excluded, even though it could offer valuable insights into community practices. Lastly, the definition of “youth” in the review was limited to the ranges available in the databases searched (youth being considered 14–25 years old), which is not aligned with the latest definitions of youth by the World Health Organisation WHO and United Nations that include youth up to 29 years old. Other limitations include that two articles (34) did not report results as they overviewed programs. These articles could therefore not contribute to program outcomes informing the creation of youth employment standards, and understanding best practices for employment support programs.

To account for the reality of ongoing community programs in Canada that may not have been captured in research databases, this project adopted a collaborative, critical pragmatic approach. The rigor was through ongoing discussion among the authors and regular meetings with and input from a larger group of interested parties. Civil society representatives and youth with disabilities provided tacit knowledge and critical insight to the entire review and analysis, enriching the scope of analysis with considerations of current practices and policies.

We also adopted an intersectional lenses framework (21) in the review, including specific terms in the search, themes in the data extraction and considerations in analysis. The results indicate that the majority of included articles limit their analysis to reporting demographic data on program participants, such as gender, age, and sex. Few studies provide deeper information on other factors, such as household income, area deprivation, or how these factors may affect youth experiences in accessing employment programs and program outcomes. Additionally, there is little consideration of how these intersectional characteristics shape the communities and systems in which the programs take place. For instance, youth with disabilities have a high prevalence of mental health conditions and the review showed that those experiencing mental health conditions have poorer employment outcomes (64). We can theorize that community mental health services should work in partnership with disability employment services to help youth navigate employment spaces and the challenges of having multiple disabilities—but this aspect was not explored at length in the studies reviewed (79).

Based on the critical gaps identified in this review, we propose several key directions for future research. The goal should be to move beyond mapping individual-level programs and toward a more explanatory, systemic, and equitable understanding of employment transitions.

1. Investigating Structural and Macro-Level Interventions: There is an urgent need for research that evaluates interventions aimed at changing systems, not just individuals. Future studies should ask: What is the impact of changes in disability policy, transportation infrastructure, or employer-focused anti-discrimination initiatives on youth employment outcomes? Methodologies such as realist evaluation of policy changes or comparative case studies of different policy environments would be valuable.
2. Adopting a Critical Intersectional Approach: Future research must integrate intersectionality as a core analytical framework. This requires both quantitative and qualitative innovation. Quantitatively, researchers can use methods like intersectional multilevel modeling to analyze how outcomes vary across multiple social positions (80). Qualitatively, participatory research methods that co-design studies with youth from diverse and multiply marginalized backgrounds can provide deep insights into their lived experiences and priorities. Key research questions include: How do the mechanisms of employment programs function differently for youth at various intersections of race, gender, class, and disability? What new mechanisms are needed to address compounded disadvantage?
3. Expanding Geographic and Contextual Diversity: To move beyond the current North American and European bias, research is needed in a wider range of global contexts, particularly in low- and middle-income countries and rural areas. This research should explore how different economic conditions, cultural norms, and policy landscapes shape employment pathways. For example, what do school-to-work transitions look like in contexts with large informal labor markets?
4. Clarifying the Contribution—From Mapping to Causal Explanation: This review serves as an explanatory map, charting the terrain of the existing evidence and, more importantly, identifying the vast uncharted territories. Future realist reviews could build on this by focusing on more specific questions to develop more refined causal explanations. For example, a future review could focus exclusively on programs that have failed, seeking to build a theory of program failure. Another could compare mechanisms across different disability types, such as visible vs. invisible disabilities, to understand how the nature of impairment interacts with social context.

By pursuing these research directions, the field can develop a more robust and equitable evidence base to inform the creation of policies and practices that support all youth with disabilities in achieving their full potential.

### 4.5 Understanding pathways to employment through contexts, mechanisms, and outcomes

To develop an understanding of how communities can create positive pathways for youth to develop in a balanced, ecological manner that may lead to employment outcomes, we must shed light onto the mechanisms that can lead to a variety of outcomes, and understand which groups of youth can benefit from each. We must also devise strategies that contribute to universal accessibility pathways.

Focusing on the interaction of micro and macro factors, literature on adult employment also reported comparable results to our review. Two studies investigating employment outcomes for adults aged 40 and over with intellectual disabilities (81) and factors to employment for adults with intellectual disabilities and autism with a median age of 33.38 (82), reported that higher education and independent living were contributive to successful employment. Our findings of the success of youth with disabilities when they received training focused on employment skills, further aligns with previous research applying Bronfenbrenner's Ecological Systems Theory. Lindsay et al. (83) emphasized the microsystem as influential to post-secondary outcomes, as well as skills of self-advocacy, communication, organization and planning as critical to successful transition.

Several studies on transition from childhood to adulthood report on the importance of support networks to achieve several positive outcomes. Zidan et al.'s realist review on interventions on transition to adulthood for youth aged 14–21 in North America (79) reported the importance of family engagement throughout health and education interventions as crucial support outlets for youth. The authors also report the importance of support for staff, receiving adequate training to tailor instruction to the needs of participants (79). Lindsay et al.'s systematic review on mentorship programs for youth with disabilities (84) reported comparable results of improvements in multiple areas such as participants' personal growth (self-efficacy, independence and social skills) for youth with disabilities who had peer-mentors. Similarly, Castruita Rios et al. (85) found that broader support networks, those increasing collaboration between stakeholders at multiple levels (employers, schools, families, service centers), when paired with activities to help youth develop their vocational skills, support for career decisions, goal setting for post-secondary education and work, were conductive with increased employment outcomes and retention (85). Support networks and related macro context factors may play a role as mechanisms and outcomes. For instance, Chen et al. (82) reported that living in areas with denser populations, higher parent household income and mothers' large social-networks were linked with employment. Other results found that employed adults with intellectual disabilities who did paid work or who were self-perceived as employed were less likely to have difficulties in social participation, and self-reported better health outcomes (81).

Findings extend through literature on broader social determinants of health. Fiorati et al. (86) found that families with better socio economic, cultural and educational conditions have better access to social settings and cultural resources, providing more opportunities for children and youth in these families to develop agency and autonomy beyond their immediate familial settings. The same authors also discuss the difficulties for study participants in lower socioeconomic status to access transportation, assistive technology and economic barriers to participate in the study intervention exposing factors that would influence access and maintenance of employment after the study. These findings point toward broader societal structures and larger familial status as a key factor in equitable access to not only employment programs, but access to healthcare services, and other community facilities (86). These C-M-Os interactions grant further exploration and purposeful inclusion of participants from different contexts and socio-economic backgrounds in programs interventions.

### 4.6 Conclusions and recommendations

This realist review sought to analyze school-to-work transition programs for youth with all disabilities with an emphasis on the contexts in which the programs run, the mechanisms adopted in each context, and their effects on the program outcomes. Findings revealed that the majority of programs incorporated elements of skills-based training related to employment tasks, social interaction and education. Programs were largely held in hospital or school settings in the United States and were mostly in urban areas. Due to the inconsistent reporting of contexts within the included studies, and the range of settings across included articles, extrapolation to gain a deeper understanding of the interactions of the micro and mesosystems mechanisms into the exo and macrosystems outcomes is limited. The gaps in macro context information point to the need to increase research that links public policies, legislation, standards and supports offered to communities as essential in the pathways to employment outcomes. Such pathways have been explored in other populations: the impact of public policies and programs on the labor market decisions of older workers (87) the impact of employment benefits (88) in Canada, and for youth in general (89).

Although Canada has a comprehensive strategy for disability employment in place (90) specific considerations for youth are lacking. In particular, an ecological approach to employment policy that considers developmental needs, and the sustainability of communities to create the right conditions for youth to develop their capacities over time, is missing. It also points to the need to investigate the variability of outcomes for different disability communities, and to highlight what community supports can lead to a variety of employment opportunities along the developmental continuum. For instance, how can we expose youth with disabilities to early vocational endeavors such as volunteering, summer jobs, community-based formal and informal employment opportunities that are often accessible for typically-developing children, and what would be the impacts of such opportunities? (91). Other consideration of macro contexts that was absent in this review is the understanding of the lived experiences of youth in rural communities, those living in territories and a variety of non-urban settings. Understanding the conditions for youth who may not be part of large programs, or where a critical mass of participants naturally leads to inclusion in mainstream employment programs, is important for developing programs, standards, and regulations that respect human rights frameworks and genuinely support universal accessibility and inclusion. Lastly, to further understandings of the diverse needs and complexities of disabled youth entering the workforce, attention must be brought to the intersectional characteristics of this population, a call that has been made for all employment groups (92). Considerations for intersectionality, and how they develop over time, from upstream perspectives are needed in future studies and in the development of youth employment programs. Studies and programs should also consider how mental health, employment, and other health and social outcomes are interconnected, following the data on Social Determinants of Health that are largely known, but scarcely applied in the development of programs on the ground (93). Further research should also consider how intersectional identities and priorities shift in communities in different geographical settings. As access to community health and social resources is context-dependent, future studies should consider how community identities intersect with community placement, and how this alters lived experience and community health. To research these intersecting identities, applying the CHILD-CHII within remote communities that specifically support individuals with intersecting social identities would allow a further comprehension of what is specifically needed to support employment transitions of youth with disabilities. This would contribute to answering research questions such as: How do intersecting identities influence the social, educational and employment outcomes for youth with disabilities? Further consideration for intersectionality beyond age, sex, gender or disability type would contribute to drive change. It can also clarify the specific mechanisms that can lead to positive outcomes for youth with disabilities who are gender diverse, who are Indigenous, who are newcomers to their country, and who may not have access to support networks in the context where they are currently living.

This review, therefore, serves as a call to action. A critical gap in the literature is the absence of research that moves beyond using intersectional categories as mere variables and instead uses an intersectional framework to guide the entire research process, from question formulation to data analysis and interpretation (94).
