# Supplementary material for: Mapping community pathways to employment for youth with disabilities—a realist review
Source: Front Public Health. 2026 Mar 17;14:1743478. doi: 10.3389/fpubh.2026.1743478 (PMC13036211; doi:10.3389/fpubh.2026.1743478)
Supplement: Supplementary file 1 [file Data_Sheet_1.docx]

Supplementary Material

# Supplementary Data

Supplementary Material should be uploaded separately on submission. Please include any supplementary data, figures and/or tables.

Supplementary material is not typeset so please ensure that all information is clearly presented, the appropriate caption is included in the file and not in the manuscript, and that the style conforms to the rest of the article.

# Supplementary Figures and Tables

**Supplemental Table 1. Data Extraction Sheet**

| **General information** |
| --- |
| Authors |
| Year of Publication |
| Title |
| Journal |
| **Study Characteristics** |
| aim of study/research question |
| **Study Participants** |
| **age** |
| number of participants |
| type of disability |
| **Intervention/recommendation** |
| what was done |
| **Context** |
| transportation |
| staff training |
| community design |
| awareness initiatives |
| healthcare access |
| general programs/services |
| volunteer/work |
| education |
| technology |
| web-mapping |
| social |
| **Accessibility Policies** |
| level (municipal, provincial/state, federal, international) |
| Who enacts the policy in question Individuals/actors/policymakers involved |
| what is the policy (guideline, act, legislation, policy recommendation - what is the policy) |
| **Intersectional considerations? (Y,N, NR, NA)** |
| gender (Y,N, NR, NA) |
| gender (descriptive) |
| sex (Y,N, NR, NA) |
| sex (descriptive) |
| immigration (Y,N, NR, NA) |
| immigration (descriptive) |
| ethnic background (Y,N, NR, NA) |
| ethnic background (descriptive) |
| indigeneity (Y,N, NR, NA) |
| indigeneity descriptive |
| **Mechanism** |
| transportation |
| staff training |
| community design |
| awareness initiatives |
| healthcare access |
| general programs/services |
| volunteer/work |
| education |
| technology |
| web-mapping |
| social |
| **Accessibility Policies** |
| level (municipal, provincial/state, federal, international) |
| Who enacts the policy in question Individuals/actors/policymakers involved |
| what is the policy (guideline, act, legislation, policy recommendation - what is the policy) |
| **Intersectional considerations?** |
| gender (Y,N, NR, NA) |
| gender (descriptive) |
| sex (Y,N, NR, NA) |
| sex (descriptive) |
| immigration (Y,N, NR, NA) |
| immigration (descriptive) |
| ethnic background (Y,N, NR, NA) |
| ethnic background (descriptive) |
| indigeneity (Y,N, NR, NA) |
| indigeneity (descriptive) |
| **Outcome** |
| Outcome |
| Results |
| Practical recommendations |
| Any outcome evaluation was done? (Y,N, NR, NA) |
| If yes descibe the evaluation |
| Strenghths of the Study |
| Limitations of the Study |

**Supplemental Table 2. Summary of included articles within their contexts, mechanisms, outcomes and Bronfenbrenner’s Ecological Systems Theory**

| **Authors, year, country, reference** | **Context (program settings and/or demographic factors when articles researching dataset)** | **Mechanism (program contents)** | **Outcome (programs' results and outcomes)** | **Program Name** | **Bronfenbrenner's Ecological Systems Theory** |
| --- | --- | --- | --- | --- | --- |
| Akinola & Doabler, 2023, United States, ([69](#_ENREF_69)). | Age (16-19 and 20-24 years), gender (male/ female), race/ethnicity (White, Black/African American, Hispanic/Latino), social security recipient status (supplementary security income and/or social security disability income recipient or non-recipient), education level (less than high school, high school diploma, special education, associate, college or graduate degree) and severity of disability (significant disability or not). | Vocational Rehabilitation guidance/counseling, assessment, assistance for job searching, work placement, supported employment, job readiness, college and occupational/vocational training, on-the-job support, job readiness training, college training, occupational/vocational training, diagnosis/treatment, maintenance and transportation service. | Educational attainment, youth being diagnosed, access to treatment services, college, on the job training, job assistance/readiness, training, supported employment, and access to guidance and counselling led to better employment outcomes and higher earnings. 63% White participants procured employment vs. 21.4% for Black/African Americans, and 14.8% for Hispanic/Latino. Employment obtention was higher for men (50.6%), and White men had higher weekly earnings, succeeded by Hispanic/Latino.participants. Transportation was not significantly contributive to employment. | State-federal Vocational Rehabilitation programs. | Microsystem (mechanism): program contents-direct support for participants. Macrosystem (context): participants' sociocultural identities (age, race/ethnicity, education level). |
| Almalki, 2021,  United States, ([27](#_ENREF_27)) | Mercy Medical Center, Midwest USA. | internship, job skills training, independent living and social skills courses. | Increase in social and vocational skills and job securement. | Project SEARCH. | Microsystem (mechanism): program contents-direct support for participants. |
| Anand & Honeycutt, 2020, United States, ([42](#_ENREF_42)) | Type of disability (mental health condition, type of Vocational Rehabilitation received, gender, age educational level, race/ethnicity, education/school enrollment status, Individualized Education Plans, Federal disability benefit status. | College support post-secondary educational support, vocational training support, Vocational Rehabilitation services different than post-education support (federal data) vocational/college training support (e.g., tuition/supply payment). | Postsecondary education was associated with successful employment. Youth receiving support with mental health considerations had lower employment rates than other disabilities. Youth with mental health conditions receiving support in school setting closed the employment gap in comparison to youth with mental health conditions receiving services in other settings.  Results not reported for age, gender, race/ethnicity, Individualized Education Plan, or school enrollment. | Multiple vocational rehabilitation programs. | Microsystem (context and mechanism): school setting and program components-direct support for participants. |
| Ashburner et al., 2018, Australia, ([64](#_ENREF_64)) | Community-based setting (vs. setting only for disabled persons). | College/post-secondary education training and support, outings to tertiary education institution, outings for social gatherings (e.g. cafés), pairings with mentors. | Program led to skills development, increase on motivation, development of understanding of future options for some difficulties with expressing goals for others. | Studio G. | Microsystem (mechanism): program contents-direct support for participants. |
| Balcazar et al., 2012, United States, ([51](#_ENREF_51)) | Summer Transition Institute, urban setting. | Preparation for college applications, vocational rehabilitation and financial services, job shadowing, job support, organization of "job clubs" for participant support with job search, follow-up job support by case managers for employment needs, support from special education staff, providing participants with access to assistive technology as needed. Interviews with case managers. | Program led to an increase in job attainment and retention, wages, and enrollment in postsecondary education for the majority of participants. Lower functioning participants worked less hours and were paid less while receiving equal or more support. Case managers assisted participants with goal setting, acquiring skills for employment (goal setting, help with job applications, etc), college (help with applications, applications to financial aid etc.), and familial support (mediation, etc.). | College Connection Program. | Microsystem (mechanism): program contents-directly involve participants.  Exosystem (mechanism): financial services involve broader structures and organizations. |
| Barnard-Brak et al., 2023, United States, ([19](#_ENREF_19)) | Collegiate program, University of Alabama. | Job placement and training catered to placement while enrolled in local educational agencies. | Job skills paired with being job-specific task analysis performance was a predictor of employment. Severity of participant's impairments, specifically in the participants intellectual and adaptive behaviors was not found to be related to employment outcome. | Crossing Points. | Microsystem (context and mechanism): school setting and school placement, direct support curriculum. Exosystem (mechanism): education services involve broader structures and organizations |
| Belisle et al., 2023, *United States,  ([43](#_ENREF_43)) | Alternative day-treatment program. | Response to photo, matching photos to job titles and responsibilities. | Improved ability to correctly match job positions to photos. | PEAK Relational Training System. | Microsystem (mechanism): program contents-directly involve participants. Macrosystem (mechanism): participants involve broader contexts to match photo to job description. |
| Benson et al., 2021, NR ([88](#_ENREF_88)) | Community-based setting. | Parent interviews on perspectives terms of transition processes. | Study reported a lack of guidance during the transitional periods, and that schools were not helpful in catering services towards their child and finding what works for them. Support received from service centers was limited and the parents were left to be the main procurers of moving the transition period foreword and achieving milestones. | Individualized education program (IEP). | Microsystem (mechanism): interviews with direct family. |
| Berastegui et al., 2023, Spain, ([34](#_ENREF_34)) | University. | University post-compulsory training program. Specific program components NR. | Moderate level of social competency development, higher increase in employment with time, after program completion. | DEMOS | Microsystem (context and mechanism): school setting and program contents-direct support for participants. |
| Black et al., 2017, United Kingdom, ([61](#_ENREF_61)) | Shopping precinct local to the Greenside School. | Travel training to and from the studio where the program is held, courses to develop communication, social and independence, social skills gatherings (public and social interaction opportunities). | Development of learning opportunities related to the curriculum, development of understandings to incorporate educational material within business setting/ understanding the bases of a business. Development of cooking, retail, travel and ceramics skills. Improvement of social skills. | Greenside Studio evaluation project. | Microsystem (context): close to school setting. Exosystem (mechanism): program contents involve broader social elements. |
| Burke-Miller et al. 2012, United States, ([54](#_ENREF_54)) | Primarily age 18-24 and 25-30) (EIDP study sites). | Supported employment, specific program components NR. | In comparison to older adults, (31 years and up), there were no differentiation in the likelihood of getting employed for youth with disabilities. | Employment Intervention Demonstration Program. | Macrosystem (context): participants' sociocultural identities. |
| Cairns et al., 2017, Australia, ([63](#_ENREF_63)) | Psychological wellbeing, mental health diagnosis, neurocognitive function, age (18-25), gender, secondary school attainment , main parent occupation and earnings, family history mental illness), distress, psychotic-like experiences, substance use (Headspace center). | Early intervention services to general, mental and vocational health and substance use issues, specific program components NR. | Being female, having a higher parent occupation status, no recent use of cannabis, and having a better memory associated with employment and higher salaries. Secondary school attainment, was associated with successful employment. | Headspace. | Microsystem (context): participants' family settings Macrosystem (context): participants' sociocultural contexts. |
| Carter et al., 2011, United States, ([56](#_ENREF_56)) | Disability (emotional/behavioral, intellectual and development), age (average 17.2), gender, race/ethnicity (European American, African American, reported other (i.e., Asian American, Latina/o, Native American Pacific Islander), employment skills, self-determination capacity, activities for career preparation (summer programs, mid-western state). | Summer employment, specific program components NR. | The majority of participants attained and maintained employment. Disability type was not found to be correlated with weekly earnings, but employment skills were found to be predictive of weekly earnings. Older youth had higher weekly earnings. Descriptive results for minority status were NR. | Federally funded development project. | Macrosystem (context): participants' sociocultural contexts |
| Cmar & McDonnall, 2019, United States, ([49](#_ENREF_49)) | Summer program, large city. | Group activities to build social support, summer job searching program, job search, job search/interview/employment support. | Increase in self-efficacy for job searching. | Putting Your Best Foot Forward. | Microsystem (mechanism): program contents-direct support for participants. |
| Cmar & McDonnall, 2021, United States, ([50](#_ENREF_50)) | Summer program, large city. | Group activities for self-efficacy, social support, job search and interview support. | No demonstration that the intervention impacted job search behavior or self-efficacy outcomes. | JOBS program | Microsystem (mechanism): program contents-direct support for participants. |
| Devine et al., 2018, United States, ([28](#_ENREF_28)) | University office. | Training for reading out loud and text comprehension using systematic prompting to deliver program. | Text comprehension levels varied amongst participants ranging from 53% to 85% of comprehension questions answered correctly. | Post-secondary education program. | Microsystem (mechanism): program contents-direct support for participants. |
| Di Rezze et al., 2023, Canada ([32](#_ENREF_32)) | University campus. | Curriculum elements of professionalism and expectations, self-advocacy, paid work with job coach support. | Improvement of social skills, self-determination, understanding the workforce. | Job-Train Program. | Microsystem (mechanism): program contents-direct support for participants. |
| Estrada-Hernandez et al., 2008, United States, ([55](#_ENREF_55)) | Severity of disability (less severe, requiring more individualized attention and accommodation, requiring considerable individualized attention) (data from program). | Vocational and on the job-training, job shadowing, development of personal plans based on personal interest to help in career guidance. | Job placements matched participants' interests. Disability severity had no effect on monthly wages. | Super Senior Program. | Microsystem (mechanism): program contents-direct support for participants. |
| Gold et al., 2013, United States, ([72](#_ENREF_72)) | age, gender, primary disability (learning, intellectual disabilities, severe emotional and behavioral disorders, Sensory Disorders combining blindness, deafness, and speech/language impairments, orthopedic, chronic health impairments, attention deficit hyperactivity disorder, autism spectrum disorders, very low prevalence disabilities), special educational services (segregated, regular and integrated into regular curriculum setttings), previous work, household income, program site (Atlanta, Chicago, Dallas, District of Columbia, Los Angeles, New Orleans, Philadelphia, Oakland, and San Francisco), and program year (following academic year), Race/ethnicity (Black , African American, Hispanic/Latino, Asian American, White, American Indian, Alaskan Native. | Job shadowing, career counselling, staff assistance within job placements. | The rate of employment from the article's program was higher than other studies of employment during high school. | Bridges Program. | Microsystem (context and mechanism): school placement and program contents-direct support for participants. Macrosystem (context): participants' sociocultural identities. |
| Hanson et al., 2021, United Kingdom, ([41](#_ENREF_41)) | Supported Internship Organization. | work, school or college work placement | Improved perceived personal capacities, improvement in work satisfaction and fulfilment and self-determination | Supported Internship | Microsystem (mechanism): program contents-direct support for participants. |
| Harun et al., 2019, ([40](#_ENREF_40)) | Secondary school graduates of education programs | Interviews with special education program graduates (vocational skills taught by instructors). | Significant associations between employment and gender, family income, the educational levels of mothers, parent expectations, financial support, vocational training and employment services. | Special Education Program. | Microsystem (mechanism): interviews with youth on personal experiences with program. |
| Honsbeger et al., 2019, Malaysia, ([38](#_ENREF_38)) | Workplace, food truck in school parking lot. | Literacy-based intervention of setting up food truck workstation/coffee station, vocation preparation. | Improved independence and employment skills. | Workplace Placement Program. | Microsystem (context and mechanism): school setting and program contents-direct support for participants. |
| Jonsson, 2021, Sweden, ([47](#_ENREF_47)) | KIND Child and Adolescent Psychiatric unit, and ADHD unit of Northern Stockholm Psychiatric Service. | Group workshops, mentorship for individual goals, group discussions on heathy lifestyles. | Participants reached levels beyond the initial set goals, a significant proportion of goals set did not follow the expected outcome, meaning that some goals did not carry through until the end. | TRANSITION program. | Microsystem (mechanism): program contents-direct support for participants. |
| Kaehne & Beyer, England 2013, ([59](#_ENREF_59)) | Workplace. | Job placement. | Improvement of social skills and independence. Interviews with parents revealed barriers include employment seen as low priority, placing importance on transition to higher education. employment options explored to fullest extent, inconsistencies in weighing funding options by all stakeholders. | Youth Supported Employment Program. | Microsystem (mechanism): program contents-direct support for participants. |
| Kiegaldie et al., 2023, Australia, ([46](#_ENREF_46)) | Hospital work setting. | Interviews with students’ supervisors on supported internship program. | Increase in communication skills, reading, technology use, problem solving as well as organization, self-determination and motivation, and an increase in employment. | Integrated Practical Placement Program. | Microsystem (mechanism): program contents-direct support for participants. |
| Lee et al., 2019, *Australia, ([62](#_ENREF_62)) | Workplace in Information and Communication Technology-related organization. | Work placement internship program, matching interests, skills and strengths. | Increase in confidence, understanding of the workforce, positive feeling towards contributing to work, society and a realization of capacities. | Autism Academy for Software Quality Assurance Integrated Program. | Microsystem (mechanism): program contents-direct support for participants. |
| Lindsay et al., 2012, Canada, ([73](#_ENREF_73)) | Children’s hospital- Ontario. | Work placement based on participants' interests. | Increase in social skills, communication and self-confidence. Increase in practical skills for employment (interview, office skills, resume writing, organizing transportation, ability to disclose disability and ask for accommodations. | Life skill's program. | Microsystem (mechanism): program contents-direct support for participants. |
| Lindsay et al., 2013, Canada, ([70](#_ENREF_70)) | Workplace- pediatric hospital. | Supported work placements, skill-building workshops, personnel to assist in self-care strategies. | Majority of youth were able to disclose their disabilities within a work context and ask for the accommodations they require. | Employment training program. | Microsystem (mechanism): program contents-direct support for participants. |
| Lombardi et al., 2017, United States, ([24](#_ENREF_24)) | Numerous schools in Connecticut. | Online career and vocational readiness and exploration, IT literacy, reading, financial literacy. | Increase for participants from 9 to 31% in knowledge of use of internet, reading, test-taking, strategies for writing, career interest, post-secondary education and training, goal setting and plan making, job preparation such as resume, interviews and soft skills. | Individualized education program (IEP). | Microsystem (context and mechanism): school setting and program contents-direct support for participants. Exosystem (mechanism): financial literacy involves broader structures and organizations. |
| Luecking et al., 2018, United States, ([23](#_ENREF_23)) | school setting Maryland. | Individualized employment experience (internship, workplace tours, summer work experiences) integrated employment (with non-disabled co-workers), family support, vocational rehabilitation case initiation coordination and collaboration between teachers, systems and staff. | Increase in employment, higher obtention of individual education plan (IEP), shorter times between processing and obtention of IEP, higher likelihood of receipt of job assistance and support, lower costs of services with MSTC model. | Maryland Seamless Transition Collaborative Model. | Microsystem (context and mechanism): school setting and program contents-direct support for participants. |
| Milbourn et al., 2020, Australia, ([57](#_ENREF_57)) | 7 metropolitan Men's Sheds. | Mentorship, opportunities to connect with peers | Increase in social skills, woodworking and use of tools. | Men’s Shed Program. | Microsystem (mechanism): program contents-direct support for participants. |
| Mlynaryk et al., 2017, Canada, ([31](#_ENREF_31)) | Specialized high school. | Job readiness, individualized tools and strategies fit for personal needs, rehabilitation services, social work support. | An active involvement of the school’s staff in activities with external partners was found as facilitator to employment transitions. | School-to-work program. | Microsystem (context and mechanism): school setting and program contents-direct support for participants. |
| Muller & VanGilder, 2014, United States, ([21](#_ENREF_21)) | Hospital, non-public school and adult service provider, Washington, D.C. | Work-site experiences, internships. | 50% of interns received employment. Increase in confidence, self-esteem, motivation and increase of understanding of work. | Project SEARCH | Microsystem (mechanism): program contents-direct support for participants. |
| Muthumbi, 2008, United States, ([20](#_ENREF_20)) | Multiple community settings, five sites in New York State, (urban areas: local work-force development consortium and independent living center and suburban/rural area : local work-force development consortium, adult provider agency, and educational agency). | Education services, education services to help with vocation support. | Increase in service awareness, increase in ability to link youth with services, increase in employer understandings of disability needs, leading to increase in skill acquisition for youth and employability. Career assessments and programs rage widely amongst schools. | Services from One-Stop Centers. | Exosystem (context): adult provider and educational agency. |
| Nicholas et al., 2019, Canada, ([60](#_ENREF_60)) | After school, workplace. | Job skills training, vocational tasks (e.g. clothes sorting in retail setting, food preparation). | Increase in job readiness skills, human interaction, confidence. | Community Works Canada. | Microsystem (context and mechanism): school setting and program contents-direct support for participants. |
| Pebdani, 2014, United States, ([48](#_ENREF_48)) | Workplaces in eight urban settings (Atlanta, Chicago, Dallas, Los Angeles, Philadelphia, San Francisco, Oakland, Washington, DC.). | Paid employment support, work placement. | Participants who had previous vocational education were more likely to terminate program participation prematurely. | Bridges From School to Work. | Microsystem (mechanism): program contents-direct support for participants. |
| Randall et al., 2020, United States, ([22](#_ENREF_22)) | University, Southeastern United States. | Teach and deliver specific tasks, providing participants with step-by-step pictures, video and audio the tasks for independent living, employment. | Increase in office-related skills such as photocopying or scanning. | Post-secondary education program, Task Analysis application. | Microsystem (context and mechanism): school setting and program contents-direct support for participants. |
| Rogan et al., 2014, United States, ([25](#_ENREF_25)) | Urban university campus, Indianapolis. | Individualized planning (goal setting, interests, careers, students hired for peer-support assistance for note taking, schoolwork, making friends, and event attendance, etc.) Multiple life-skills opportunities (e.g. familiarization/navigation of transportation routes, time management, internships, academic skills training, etc.). Use of on-campus computers to update journals. Web mapping used to link services/programs on website. | Employment rates vary year to year for participants, improvement of academics, computer skills, self-determination skills, social skills, career preparation and independence. | Skills for Independence, Transition and Employment SITE) program. | Microsystem (context and mechanism): school setting and program contents-direct support for participants. |
| Rumrill et al., 2016, *United States, ([45](#_ENREF_45)) | Age (16-25), gender, race/ethnicity (White, Black or African American, Hispanic or Latino, Asian or Pacific Islander, American Indian or Alaska Native) , depression/mood disorders education level at time-of-service enrollment, disability benefits . | Diagnosis, health services, job support, educational attainment data, counselling, job placement and retention services, vocational training. | Individuals who were successfully employed received more services, vocational services such as supplemental security income and on the job placements and less time in rehabilitation. Educational and receipt of disability benefits associated with successful employment. Age, gender and race/ethnicity was not associated with successful employment. | Vocational rehabilitation services. | Microsystem (mechanism): program contents-direct support for participants. Macrosystem (context): participants' sociocultural identities. Exosystem (mechanism): health services involve broader structures and organizations. |
| Scanlon & Doyle, 2021, Ireland, ([39](#_ENREF_39)) | Two special schools. | Personalized progression plans with the support of a career facilitator, post-school planning. | Increase in confidence and certainty about planning for the future. | Walkinstown Association for People with an Intellectual Disability (WALK) Program. | Microsystem (context and mechanism): school setting and program contents-direct support for participants. |
| Schall et al., 2020, United States, ([71](#_ENREF_71)) | four hospitals, Virginia. | Internship program, support by educational staff. | Increase in employment rate, social skills, higher self-management and technology use skills. | Project SEARCH. | Microsystem (mechanism): program contents-direct support for participants. |
| Schillaci et al., 2021, United States, ([26](#_ENREF_26)) | College, Massachusetts. | Individualized planning, internship/work opportunities, learning plans, mentor/education/work support, access to accommodations, support through education coaches, access to disability services. | Improvement of self-initiation and volitional action autonomy, independence, self-determination. | Think College Transition project. | Microsystem (context and mechanism): school setting and program contents-direct support for participants. Exosystem (mechanism): disability services involve broader structures and organizations. |
| Schlegelmilch et al., 2021, United States, ([65](#_ENREF_65)) | Multiple community/service settings, Wisconsin. | Financial coaching, advocacy, family advocacy, health support/health promotion training, soft skills training. | Increase in confidence and positive outlook for youth’s futures. Services for the whole family is key to successful employment. For one participant, their medical condition (physical disability, deafness, blindness and autism) was a barrier to accessing employment and the services offered by the program, although enrollment in the program made them want to work, changing their perspectives. | WI PROMISE. | Microsystem (mechanism): program contents-direct support for participants. Exosystem (mechanism): financial services involve broader structures and organizations. |
| Shatil et al., 2023, Bangladesh, ([37](#_ENREF_37)) | School. | Apprenticeship, classroom training, work support, topics of employer-employee workplace relationship, social issues and stigmas, workplace activities. | Employment rate for disabled women and girls is lower than disabled men facing more severe social exclusion and being more vulnerable to sexual harassment, strong monitoring and to multiple discriminations. | Skills Training for Advancing Resources (STAR) Program | Microsystem (context and mechanism): school setting and program contents-direct support for participants. |
| Skellern & Astbury, 2014, United Kingdom, ([36](#_ENREF_36)) | College. | Work and life skills courses. | Increase in career preparation, independence and confidence. | Further education program. | Microsystem (context and mechanism): school setting and program contents-direct support for participants. |
| Strater & Elfers, 2019, United States, ([53](#_ENREF_53)) | Kentucky distribution center. | Internship, public and social interaction opportunities, setting employment goals. | Increase in self-determination and development of goals set by the participants, increase in job securement, improvements of work independence. | Project SEARCH. | Microsystem (mechanism): program contents-direct support for participants. |
| Strickland et al., 2013, United States, ([30](#_ENREF_30)) | Web-program, Emory university. | Quizzes, and scenarios, supporting youth in learning interview skills through technology. | Development of interview skills. | JobTIPS program | Microsystem (mechanism): program contents-direct support for participants. |
| Traina et al., 2022, Ireland, ([35](#_ENREF_35)) | University, and disability service provide, | Employment preparation, individualized employment placement, teaching about living healthily, familiarization/navigation of transportation routes. | Improvements of social and communication skills, independent living time management, colleague interactions. | Education and training for Acquiring Employment Skills (E-IDEAS) curriculum. | Microsystem (context and mechanism): school setting and program contents-direct support for participants. |
| Trainor et al., 2008, United States, ([52](#_ENREF_52)) | Summer workplace, other community settings. | Interview of teachers on their perceptions of summer transition program, summer work, internships, work experience. | Curriculums don't focus on what certain youth need, (e.g programs not going beyond topics of functional or academic skills.) Inclusion criteria for programs may exclude participants based on severity of disability, (e.g. excluding those with less severe disabilities, excluding by meeting the requirement of missing credits for program eligibility). | Transition programs. | Microsystem (mechanism): program contents-direct support for participants. |
| Versnel et al., 2008, Canada, ([33](#_ENREF_33)) | Neighborhood garages. | Co-operative workplace learning (collaboration between school and workplace for work placement). | Workplace not effectively supporting students in the way they needed, workplaces where internship held and participants not adequately prepared for work placement, expectations of supervisors not met by the participants, participants unaware of their rights and how to advocate for the accommodations they required. | high-school co-operative education program. | Microsystem (mechanism): program contents-direct support for participants. |
| Vigna et al., 2023, Wales, ([58](#_ENREF_58)) | Previous work/paid employment, disability (intellectual, learning difficulty and/or autism), school/life difficulties/experiences, special needs status at school, aspirations/employment preferences. | Job placement, support with interviews, benefit planning and travel training for work, vocational assessment, career counselling. | Increase in job securement. Youth with learning disabilities, and youth intellectual disability, have a employment rate is 23%, however, if youth with autism also have a learning disability, the employment rate is 22%. When youth have autism and intellectual disability the rate is 16%. Previous work experience helps in attaining employment (24.7% vs 8.2%).  Work experience of significant duration significantly affects ability to attain work later on (37% employment with significant work vs. 16% employed without).  Youth with learning disability more likely to obtain significant employment vs. those with autism and intellectual disabilities. No difference of significant employment with age and gender. | Engage to Change Project. | Microsystem (mechanism): program contents-direct support for participants. Exosystem (mechanism): benefit planning, involves external systems. |
| Wehman et al., 2014, United States, ([67](#_ENREF_67)) | Suburban hospital, Richmond, Virginia. | Job internship, travel training to and from work. | Increase in work independence and employment. | Project SEARCH. | Microsystem (mechanism): program contents-direct support for participants. |
| Wehman et al., 2012, United States, ([44](#_ENREF_44)) | Suburban hospital. | Job internship, job skills training. | Increase in job securement. | Project SEARCH. | Microsystem (mechanism): program contents-direct support for participants. |
| Wehman et al., 2017, ([66](#_ENREF_66)) | Hospital | Job internship, support throughout internship transportation training (reading bus schedules, personal transportation planning), staff training for program staff in implementing support for participants, awareness raising for employers, social skills training, work with mentor to determine employment goals and perform assessments, complete interviews until employment was obtained, on site-training and on job support. Use of technology through program (e.g for instruction and intervention). | Increase in hours worked even a year after program completion, support intensity needed for participants throughout the program decreased, increase in wages even a year after program completion. | Project SEARCH. | Microsystem (mechanism): program contents-direct support for participants. |
| Wehman et al., 2019, United States, ([68](#_ENREF_68)) | 4 hospitals, Virginia, USA | Job internship, access to special education teachers at the same time as receiving adult services, use of technology to facilitate the preparation of a presentation for participants not able to verbally communicate, engagement of family members of participants in discussions for how employment will impact Medicaid, family, transportation, SSI, and other benefits. | The majority of participants obtained employment, higher wages and hours worked overtime. | Project SEARCH. | Microsystem (mechanism): program contents-direct support for participants. Exosystem (mechanism): adult services involve broader structures and organisations |
| Wilson et al. 2017, United States, ([29](#_ENREF_29)) | Delgado Community College, urban campus, workplace. | Providing opportunities to earn degrees, paid apprenticeship, self-determination training, classes on using transportation independently. | No reported results, article overviews a program. | PAY Check. | Microsystem (context and mechanism): school setting and program contents-direct support for participants. |
| * when program country NR, first author’s country of affiliation given | | | | | |

**Supplemental Table 3. Participant characteristics**

| **Authors, year, country, reference** | **sample size** | **age range** | **type of disability** | **sex** | **ethnicity** | **indigeneity** |
| --- | --- | --- | --- | --- | --- | --- |
| Akinola & Doabler, 2023, United States, ([69](#_ENREF_69)). | Sample of 4,772. | 16-24 | Depressive disorder. | 51.1% male, 49.9% female. | 63.8% White, 21.4% Black/African American, 14.8% Hispanic/Latino. | % of Native American, Alaskan Native, Native Hawaiian and other Pacific Islander too small to analyze. |
| Almalki, 2021,  United States, ([27](#_ENREF_27)) | NA. | 18-21 | Intellectual and developmental. | All female. | NR. | NR. |
| Anand & Honeycutt, 2020, United States, ([42](#_ENREF_42)) | NR. | 16-24 | Mental health conditions. | NR. | NR. | NR. |
| Ashburner et al., 2018, Australia, ([64](#_ENREF_64)) | 11 | 17-21 | Autism spectrum disorder, autistic disorder or asperger disorder. | 8 male 3 female youth, 3 male 9 female parents, 6 male 1 female mentors. | NR. | NR. |
| Balcazar et al., 2012, United States, ([51](#_ENREF_51)) | 164 graduates, 26 comparison group. | 18-25 | Learning, emotional, cognitive, sensory impairments, other. | 52.4% male 47.6% female. | 48.5% Latino, 44.8% African American, 4.9% White, 1.1 unspecified. | NR. |
| Barnard-Brak et al., 2023, United States, ([19](#_ENREF_19)) | 56 | average 21.91. | Intellectual, autism spectrum disorder, physical and orthopedic, traumatic brain injury, learning. | 37% female, 63% male. | 61% African American, 2% Asian American, 37% White, 2% Hispanic. | NR. |
| Belisle et al., 2023, *United States,  ([43](#_ENREF_43)) | 1 | 19 | Autism spectrum disorder, bipolar disorder, schizophrenia. | All male. | NR. | NR. |
| Benson et al., 2021, NR ([88](#_ENREF_88)) | 11 | 16-22 | Intellectual and developmental. | All female (parents), 8 female, 3 male (youth). | NR. | NR. |
| Berastegui et al., 2023, Spain, ([34](#_ENREF_34)) | 142 program alumni. | Mean age 23.48. | Intellectual. | 50.9% female, 49.1% men. | NR. | NR. |
| Black et al., 2017, United Kingdom, ([61](#_ENREF_61)) | 21, 2 youth. | 16-19 | Severe learning disabilities. | NR. | NR. | NR. |
| Burke-Miller et al. 2012, United States, ([54](#_ENREF_54)) | 1,272 | 18-24 | Psychiatric. | 39.5% female. | 34.6% African American, 22.5% Hispanic/Latino. | NR. |
| Cairns et al., 2017, Australia, ([63](#_ENREF_63)) | 107 | 18–25 | Mental health diagnosis. | 38% male. | NR. | NR. |
| Carter et al., 2011, United States, ([56](#_ENREF_56)) | 220 | Average 17.2. | EBD, intellectual, learning. | 19.7% female, 80.3% male. | 19.7% African American, 78.8% European American, 1.5% missing. | 0% Native American, 0% Pacific Islander/Hawaiian. |
| Cmar & McDonnall, 2019, United States, ([49](#_ENREF_49)) | 42 | 15-22 | Visual impairments. | 54.8% female. | 54.8% African American, 33.3% White, 2.4% Asian American, 2.4% mixed race/multiracial, 2.4% Hispanic. | NR. |
| Cmar & McDonnall, 2021, United States, ([50](#_ENREF_50)) | 92 | 15-22 | Visual impairments. | 52.2% female. | 48.9% White, 43.5% African American, 4.4% multi race/mixed race, 7% Hispanic. | NR. |
| Devine et al., 2018, United States, ([28](#_ENREF_28)) | 3 | 18-19 | Autism, down syndrome. | 1 female, 2 male. | All White. | NR. |
| Di Rezze et al., 2023, Canada ([32](#_ENREF_32)) | 12 | 15-18 | Attention deficit disorder, dyslexia, developmental coordination disorder, generalized anxiety disorder. | 10 male 2 female. | NR. | NR. |
| Estrada-Hernandez et al., 2008, United States, ([55](#_ENREF_55)) | 115 | 18-21 | Physical, cognitive, behavioral. | 29% female, 71% male. | 78.3% Caucasian/White, 10.4% African American, 5.3% Asian American, 0.7% Hispanic. | 5.3% American Indian. |
| Gold et al., 2013, United States, ([72](#_ENREF_72)) | 5,847 | 16-19 | Learning, intellectual, severe emotional and behavioral disorders, blindness, deafness, speech/language impairment, orthopedic impairments, chronic health impairments, attention deficit hyperactivity disorder, autism spectrum disorders, other very low prevalence disabilities. | 79% male, 74.4% female. | 76.9% Black, 79% Hispanic, 77.1% White, 72.3% Asian, 74.9% other. | NR. |
| Hanson et al., 2021, United Kingdom, ([41](#_ENREF_41)) | 14 | 16-24 | Learning. | NR. | NR. | NR. |
| Harun et al., 2019, ([40](#_ENREF_40)) | 90 | 18-25 | Learning. | 71.4% male, 28.6% female. | NR. | NR. |
| Honsbeger et al., 2019, Malaysia, ([38](#_ENREF_38)) | 4 | 19-22 | Autism spectrum disorder. | Three young adult females and a young adult male. | NR. | NR. |
| Jonsson, 2021, Sweden, ([47](#_ENREF_47)) | 26 | 17-24 | Autism spectrum disorder, attention deficit hyperactivity disorder. | NR. | NR. | NR. |
| Kaehne & Beyer, England 2013, ([59](#_ENREF_59)) | 5 | 17-24 | Intellectual. | 1 female, 4 male. | NR. | NR. |
| Kiegaldie et al., 2023, Australia, ([46](#_ENREF_46)) | 55 intervention, 38 control. | mean age 19 | Intellectual, learning difficulty, autism spectrum disorder, hearing or vision impaired, physical, down syndrome. | Intervention 50% male 50% female, Comparison 56.3% male 40.6% female. | NR. | NR. |
| Lee et al., 2019, *Australia, ([62](#_ENREF_62)) | 17 | 15-18 | Autism spectrum disorder. | 6 female parents, adolescents male. | NR. | NR. |
| Lindsay et al., 2012, Canada, ([73](#_ENREF_73)) | 18 | 15-21 | Physical disabilities. | 8 male 8 female. | NR. | NR. |
| Lindsay et al., 2013, Canada, ([70](#_ENREF_70)) | 18 | 15-20 | Physical and/or mobility. | 9 males, 9 females. | NR. | NR. |
| Lombardi et al., 2017, United States, ([24](#_ENREF_24)) | 108 | Mean 17.32. | Learning, ADHD, autism, emotional disturbance, intellectual, speech and language. | 32 male, 32 female. | 13 Hispanic, 51 Non-Hispanic. | NR. |
| Luecking et al., 2018, United States, ([23](#_ENREF_23)) | 377 | Mean 17.615. | Psychiatric/serious emotional disturbance, specific learning, autism. | 29.4% female. | 60.7% Non-Hispanic White, 36.1% Non-Hispanic Black. | NR. |
| Milbourn et al., 2020, Australia, ([57](#_ENREF_57)) | 18 | 17-24 | Intellectual. | Men, family members 4 female mothers, one father. | NR. | NR. |
| Mlynaryk et al., 2017, Canada, ([31](#_ENREF_31)) | 100 | 18-21 | Moderate to severe physical disabilities. | Sample characteristics 4 men 2 female. | NR. | NR. |
| Muller & VanGilder, 2014, United States, ([21](#_ENREF_21)) | 10 | 17-24 | Learning, cognitive, autism. | 6 male 4 female. | NR. | NR. |
| Muthumbi, 2008, United States, ([20](#_ENREF_20)) | NR. | 18-24 | NR. | NR. | NR. | NR. |
| Nicholas et al., 2019, Canada, ([60](#_ENREF_60)) | 76 | 15-22 | Autism spectrum disorder. | 85% male participants, 75% female peer mentors. | NR. | NR. |
| Pebdani, 2014, United States, ([48](#_ENREF_48)) | 6,227 | 16-24 | Learning, developmental, emotional/behavioral, sensory, physical, other. | 58.7% male, 41.3% female. | 63% African American, 22.4% Hispanic, 8.4% White, 2.9% Asian, 3.1% other. | NR. |
| Randall et al., 2020, United States, ([22](#_ENREF_22)) | 4 | 19-20 | Intellectual. | All female. | NR. | NR. |
| Rogan et al., 2014, United States, ([25](#_ENREF_25)) | 12 | 18-21 | Intellectual. | NR. | NR. | NR. |
| Rumrill et al., 2016, *United States, ([45](#_ENREF_45)) | 1546 | 16-25 | Traumatic brain injury. | 32.8% female, 67.2% male. | 72.6% White, 13.1% African American, 9.6% Hispanic/Latino, 2.5% Asian. | 2.1% American Indian. |
| Scanlon & Doyle, 2021, Ireland, ([39](#_ENREF_39)) | 31 | 16-21 | Intellectual. | NR. | NR. | NR. |
| Schall et al., 2020, United States, ([71](#_ENREF_71)) | 156 | 18-24 | Autism spectrum disorder. | Treatment participants: 72% male control: 83% male control dropouts: 74% male. | 57% White, 43% non-White (African American, Hispanic, Asian). | NR. |
| Schillaci et al., 2021, United States, ([26](#_ENREF_26)) | 67 | 18-22 | Intellectual. | Intervention group: 64% male. | 75% White/Caucasian, 14% Hispanic/Latino, less than 5 % African American or Black, <5% Asian, <5% other. | NR. |
| Schlegelmilch et al., 2021, United States, ([65](#_ENREF_65)) | 1,012 | 14-16 | Physical, cognitive impairments, deaf, blind, autism. | NR. | %NR. | NR. |
| Shatil et al., 2023, Bangladesh, ([37](#_ENREF_37)) | 35 | Average age male 19 female 19.4. | Speech, physical, vision, intellectual, hearing. | 24 male, 10 female. | NR. | NR. |
| Skellern & Astbury, 2014, United Kingdom, ([36](#_ENREF_36)) | 23 | 16-25 | Learning. | NR. | NR. | NR. |
| Strater & Elfers, 2019, United States, ([53](#_ENREF_53)) | 9 | 18-20 | Intellectual. | NR. | NR. | NR. |
| Strickland et al., 2013, United States, ([30](#_ENREF_30)) | 22 | 16-19 | High functioning autism spectrum disorder, asperger disorder. | 100% male. | Control group: 27.3% African American, 63.6% White, 9.1% Biracial intervention group: 18.2% African American, 72.7% White, 9.1% other. | NR. |
| Traina et al., 2022, Ireland, ([35](#_ENREF_35)) | 5 | 19-22 | Intellectual. | 2 male, 3 female. | NR. | NR. |
| Trainor et al., 2008, United States, ([52](#_ENREF_52)) | 14 | Teachers of youth aged 18 to 21. | Cognitive, autism, multiple disabilities. | NR. | European American, African American, Latino, and Asian American backgrounds. | NR. |
| Versnel et al., 2008, Canada, ([33](#_ENREF_33)) | 2 | 16-18 | Learning. | NR. | NR. | NR. |
| Vigna et al., 2023, Wales, ([58](#_ENREF_58)) | 1008 | 16-25 | Intellectual, learning, autism. | 75% male. | NR. | NR. |
| Wehman et al., 2014, United States, ([67](#_ENREF_67)) | 40 | 18-21 | Autism, pervasive developmental disorder-not otherwise specified, asperger. | Control group: 68% male treatment group: 75% male. | Control group: 46.7 % African American 46.7 % White 6.7 % Asian  treatment group: 41.7 % African American 58.3 % White 0 % Asian. | NR. |
| Wehman et al., 2012, United States, ([44](#_ENREF_44)) | 2 | 18-22 | Autism spectrum disorder. | NR. | NR. | NR. |
| Wehman et al., 2017, ([66](#_ENREF_66)) | 54 | 18-21 | Autism spectrum disorder. | (77.4%) male, (22.5%) female. | (38.7%) African American, (58.1%) Caucasian, (3.2%) Asian. | NR. |
| Wehman et al., 2019, United States, ([68](#_ENREF_68)) | 156 | 18-21 | Autism spectrum disorder. | 72% male, 28% female. | 57% White, 43% Non-White. | NR. |
| Wilson et al. 2017, United States, ([29](#_ENREF_29)) | 12 | 18-21 | NR. | Half of sample characteristics male. | NR. | NR. |
| * when program country NR, first author’s country of affiliation given | | | | | | |

## Supplemental Figure 1. Search strategy

##
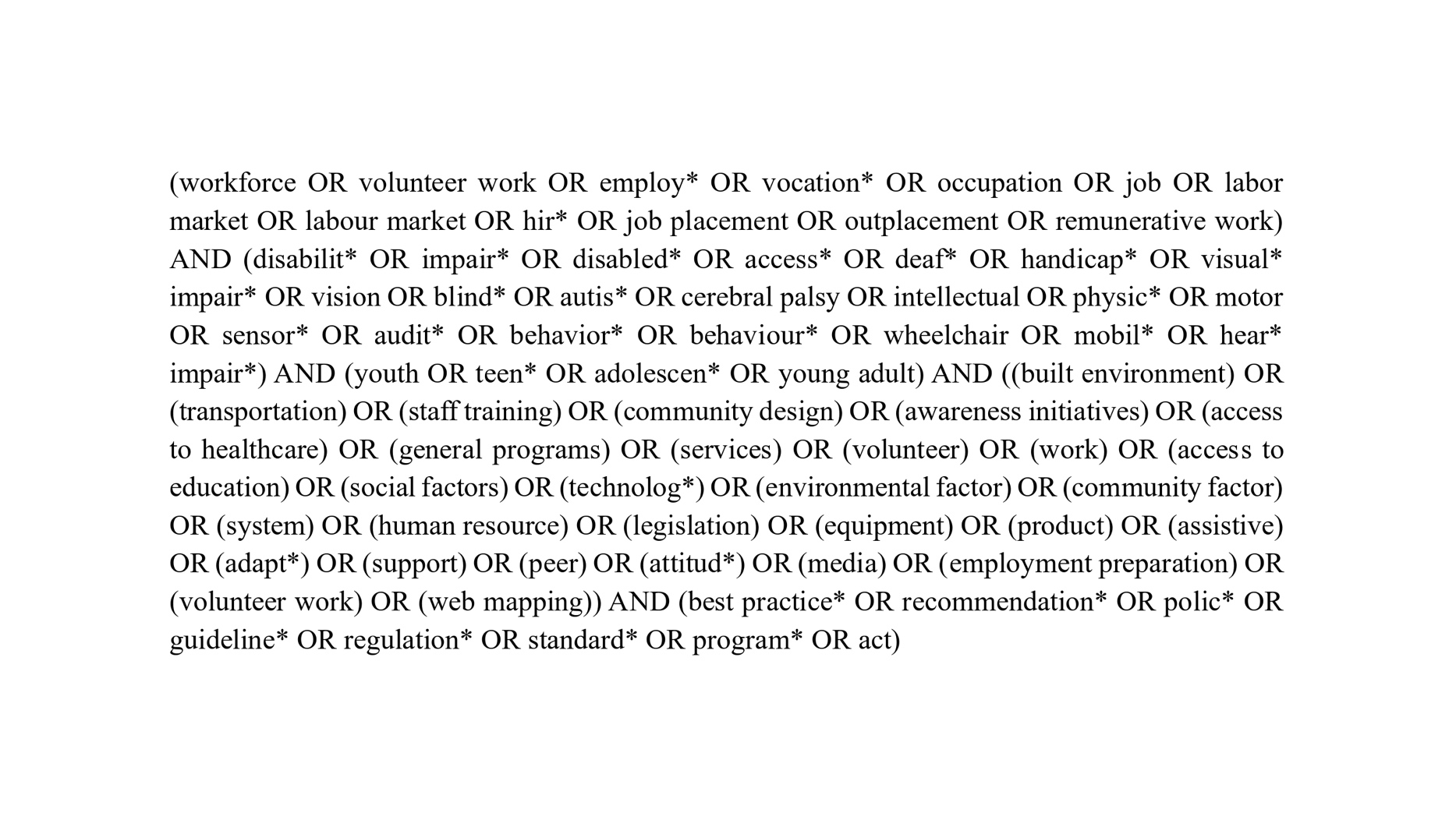


## Supplementary File 1. Search strategy for all databases

| Database | Medline |
| --- | --- |
| Interface | Ovid |
| Date | April 25^th^ 2024 |
| Limits | Date: 2008-Current |
| Number of Results | 6638 |

**1**  Adolescent/ or Young Adult/ (2712733)
**2**  (youth* or adolescen* or teen*).tw,kw. (470933)
**3**  (young* adj2 (people* or adult*)).tw,kw. (185373)
**4**  exp Occupational Groups/ (740944)
**5**  employment/ or career mobility/ or employment, supported/ or unemployment/ or job security/ or return to work/ (73214)
**6**  (employ* or work force* or workforce* or work or job* or occupation* or labo?r or re-employ* or reemploy* or unemploy* or un-employ* or vocation*).tw,kw. (2415636)
**7**  Community Integration/ (448)
**8**  (communit* adj2 (service* or support* or integration* or factor* or resource* or system* or policy or policies or standard* or program* or assist* or design*)).tw,kw. (57279)
**9**  Volunteers/ (11012)
**10**  volunteer*.tw,kw. (221830)
**11**  teleworking/ or workplace/ (30808)
**12**  (telework* or "work from home").tw,kw. (2696)
**13**  (work* adj2 remote*).tw,kw. (1695)
**14**  (workplace* or work place* or work environment* or work space* or workspace*).tw,kw. (75602)
**15**  legislation/ (1676)
**16**  ((government* or legislat* or provinc* or municip* or federal* or national or organization* or organisation* or "not for profit" or "for profit" or nonprofit or non profit or association*) adj2 (support* or assist* or policy or policies)).tw,kw. (49527)
**17**  Environment Design/ (7322)
**18**  ((environment* or facility or facilities or building* or city or cities or urban) adj2 (design* or construct* or plan* or configur*)).tw,kw. (22370)
**19**  exp "Facility Design and Construction"/ (26300)
**20**  Built Environment/ (1415)
**21**  built environment*.tw,kw. (6262)
**22**  (sidewalk* or road* or street* or ramp*).tw,kw. (106021)
**23**  (transportation or paratransit or para transit).tw,kw. (48104)
**24**  ((specially-designed or assistive or adaptive) adj2 (technolog* or equipment or product*)).tw,kw. (4209)
**25**  exp Health Services Accessibility/ (136649)
**26**  access*.tw,kw. (728482)
**27**  aware*.tw,kw. (320372)
**28**  ((staff* or personnel* or workforce*) adj2 (train* or practic* or instruct*)).tw,kw. (22548)
**29**  (therap* adj2 (social club* or support group* or organi?ation*)).tw,kw. (721)
**30**  ((peer* or group* or famil* or friend* or caregiver*) adj2 (support* or assistance or aid)).tw,kw. (52986)
**31**  (social adj2 (activit* or gathering* or factor*)).tw,kw. (34019)
**32**  Attitude to Health/ (85467)
**33**  (attitude* or perception*).tw,kw. (519376)
**34**  Social Perception/ or Public Opinion/ or Social Stigma/ or Stereotyping/ or Social Discrimination/ or exp Social Environment/ (192781)
**35**  ((social or societ* or communit*) adj3 (condition* or environment* or context* or attitude* or inclus* or support*)).tw,kw. (154936)
**36**  (stereotyp* or discriminat* or stigma* or acceptance or inclusi*).tw,kw. (866293)
**37**  Developmental Disabilities/ (22597)
**38**  exp Disabled Persons/ (74765)
**39**  (disabilit* or disabled or impaired or impairment* or handicap*).tw,kw. (1053060)
**40**  (deaf* or ((hearing or audit*) adj2 (impair* or handicap*))).tw,kw. (58974)
**41**  "hard of hearing".tw,kw. (1917)
**42**  (blind* or ((visual or ocular or vision) adj2 (handicap* or impair*))).tw,kw. (380352)
**43**  Autistic Disorder/ (26508)
**44**  (autism or autistic or asperger*).tw,kw. (70764)
**45**  brain injury, chronic/ or cerebral palsy/ (25031)
**46**  (cerebral palsy or spastic diplegia or little disease or little's disease).tw,kw. (28555)
**47**  exp Wheelchairs/ (5521)
**48**  (wheelchair* or mobility device* or mobility scooter* or mobility aid*).tw,kw. (9791)
**49**  1 or 2 or 3 (2906446)
**50**  4 or 5 or 6 (3043083)
**51**  or/7-36 (3108698)
**52**  or/37-48 (1545268)
**53**  49 and 50 and 51 and 52 (8598)
**54**  limit 53 to yr="2008 -Current" (6638)

| Database | Google Scholar |
| --- | --- |
| Interface | Harzing's Publish or Perish (macOS GUI edition)  8.12.4612.8838 (2024.03.12.1311) |
| Date | April 25^th^ 2024 |
| Limits | Date: 2008-2024 |
| Number of Results | First 200 |

(adolescent|teen|youngadult)(work|job|employment)(society|environment|barrier|faciliator|attitude|legislation)(disabled|deaf|blind|autism|"cystic fibrosis")

| Database | PsycInfo |
| --- | --- |
| Interface | Ovid |
| Date | April 25^th^ 2024 |
| Limits | Date: 2008-Current |
| Number of Results | 3750 |

**1**  (Adolescent or Young Adult).mh. (364230)
**2**  (youth* or adolescen* or teen*).tw. (392030)
**3**  (young* adj2 (people* or adult*)).tw. (112551)
**4**  Occupational Groups.mh. (0)
**5**  exp Occupations/ (74995)
**6**  Supported Employment/ or Disabled Personnel/ (2438)
**7**  (employment or career mobility or employment, supported or unemployment or job security or return to work).mh. (13485)
**8**  (employ* or work force* or workforce* or work or job* or occupation* or labo?r or re-employ* or reemploy* or unemploy* or un-employ* or vocation*).tw. (956656)
**9**  Community Integration.mh. (155)
**10**  Community Services/ or Community Involvement/ or Community Advocacy/ (25451)
**11**  (communit* adj2 (service* or support* or integration* or factor* or resource* or system* or policy or policies or standard* or program* or assist* or design*)).tw. (41285)
**12**  Volunteers.mh. (1484)
**13**  Volunteers/ (6276)
**14**  volunteer*.tw. (45055)
**15**  Teleworkers/ (183)
**16**  (teleworking or workplace).mh. (6270)
**17**  (telework* or "work from home").tw. (2675)
**18**  (work* adj2 remote*).tw. (1078)
**19**  (workplace* or work place* or work environment* or work space* or workspace*).tw. (62568)
**20**  Workplace Intervention/ or exp Working Conditions/ (36751)
**21**  legislation.mh. (0)
**22**  Laws/ or exp Disability Laws/ or Discrimination Laws/ (20648)
**23**  ((government* or legislat* or provinc* or municip* or federal* or national or organization* or organisation* or "not for profit" or "for profit" or nonprofit or non profit or association*) adj2 (support* or assist* or policy or policies)).tw. (29617)
**24**  Environment Design.mh. (1908)
**25**  exp Environmental Planning/ (9140)
**26**  ((environment* or facility or facilities or building* or city or cities or urban) adj2 (design* or construct* or plan* or configur*)).tw. (8324)
**27**  "Facility Design and Construction".mh. (326)
**28**  Built Environment.mh. (102)
**29**  Community Development/ (2528)
**30**  built environment*.tw. (2867)
**31**  (sidewalk* or road* or street* or ramp*).tw. (38036)
**32**  (transportation or paratransit or para transit).tw. (10471)
**33**  ((specially-designed or assistive or adaptive) adj2 (technolog* or equipment or product*)).tw. (3197)
**34**  Health Services Accessibility.mh. (11513)
**35**  exp Health Care Access/ (10484)
**36**  access*.tw. (189327)
**37**  aware*.tw. (160436)
**38**  ((staff* or personnel* or workforce*) adj2 (train* or practic* or instruct*)).tw. (10624)
**39**  (therap* adj2 (social club* or support group* or organi?ation*)).tw. (709)
**40**  ((peer* or group* or famil* or friend* or caregiver*) adj2 (support* or assistance or aid)).tw. (49112)
**41**  (social adj2 (activit* or gathering* or factor*)).tw. (33570)
**42**  "Attitude to Health".mh. (25524)
**43**  (attitude* or perception*).tw. (624808)
**44**  (Social Perception or Public Opinion or Social Stigma or Stereotyping or Social Discrimination or Social Environment).mh. (49616)
**45**  Community Attitudes/ or Public Opinion/ or exp social discrimination/ or Stereotyped Attitudes/ (46501)
**46**  exp Social perception/ (90120)
**47**  ((social or societ* or communit*) adj3 (condition* or environment* or context* or attitude* or inclus* or support*)).tw. (179931)
**48**  (stereotyp* or discriminat* or stigma* or acceptance or inclusi*).tw. (369296)
**49**  Developmental Disabilities.mh. (6310)
**50**  exp Developmental disabilities/ or exp Disabilities/ (77426)
**51**  Disabled Persons.mh. (9426)
**52**  (disabilit* or disabled or impaired or impairment* or handicap*).tw. (391113)
**53**  (deaf* or ((hearing or audit*) adj2 (impair* or handicap*))).tw. (26247)
**54**  Deaf Blind/ (353)
**55**  "hard of hearing".tw. (2815)
**56**  (blind* or ((visual or ocular or vision) adj2 (handicap* or impair*))).tw. (68977)
**57**  Autistic Disorder.mh. (11787)
**58**  exp Autism Spectrum Disorders/ (58351)
**59**  (autism or autistic or asperger*).tw. (70600)
**60**  (brain injury, chronic or cerebral palsy).mh. (3983)
**61**  Brain damage/ or Brain Injuries/ (19626)
**62**  (cerebral palsy or spastic diplegia or little disease or little's disease).tw. (8823)
**63**  Cerebral palsy/ (6505)
**64**  Wheelchairs.mh. (508)
**65**  (wheelchair* or mobility device* or mobility scooter* or mobility aid*).tw. (2289)
**66**  exp mobility aids/ (1380)
**67**  1 or 2 or 3 (721057)
**68**  or/4-8 (969545)
**69**  or/9-48 (1614980)
**70**  or/49-66 (543700)
**71**  67 and 68 and 69 and 70 (5196)
**72**  limit 71 to yr="2008 -Current" (3750)

| Database | Rehab Data |
| --- | --- |
| Interface | N/A |
| Date | April 25^th^ 2024 |
| Limits | Date: 2008-Current |
| Number of Results | 65 |

**Current Search**: Articles, not including International Research, containing at least one of the word(s): ' disable OR disability OR autism OR "asperger*" OR deaf OR blind OR palsy OR wheelchair OR "handicap*" ', where Abstract contains: youth, OR Abstract contains: adolesc*, AND Abstract contains: workforce, OR Abstract contains: employ*, and Year Published is between 2008 and 2024

https://www.naric.com/content/cf-rehab-adv-srch-redirect?search_id=394223

| Database | ERIC |
| --- | --- |
| Interface | Ebsco |
| Date | April 29^th^ 2024 |
| Limits | Date: 2008-Current |
| Number of Results | 1097 |

Top of Form

|  |
| --- |

| **#** | **Query** | **Results** |
| --- | --- | --- |
| S22 | S4 AND S15 AND S16 AND S21 | 1,097 |
| S21 | S17 OR S18 OR S19 OR S20 | 136,480 |
| S20 | DE "Assistive Technology" | 3,746 |
| S19 | TI ( disabilit* or disabled or impaired or impairment* or handicap* or "hard of hearing" or autism or autistic or asperger* or cerebral palsy or spastic diplegia or little disease or little's disease or wheelchair* or mobility device* or mobility scooter* or mobility aid* ) OR AB ( disabilit* or disabled or impaired or impairment* or handicap* or "hard of hearing" or autism or autistic or asperger* or cerebral palsy or spastic diplegia or little disease or little's disease or wheelchair* or mobility device* or mobility scooter* or mobility aid* ) OR TI ( (deaf* or ((hearing or audit*) n2 (impair* or handicap*))) ) OR AB ( (deaf* or ((hearing or audit*) n2 (impair* or handicap*))) ) OR TI ( (blind* or ((visual or ocular or vision) n2 (handicap* or impair*))) ) OR AB ( (blind* or ((visual or ocular or vision) n2 (handicap* or impair*))) ) | 129,510 |
| S18 | DE "Deaf Blind" OR DE "Blindness" | 4,253 |
| S17 | DE "Intellectual Disability" OR DE "Down Syndrome" OR DE "Mild Intellectual Disability" OR DE "Moderate Intellectual Disability" OR DE "Severe Intellectual Disability" OR DE "Autism Spectrum Disorders" OR DE "Cerebral Palsy" OR DE "Developmental Disabilities" OR DE "Neurological Impairments" OR DE "Aphasia" OR DE "Cerebral Palsy" OR DE "Epilepsy" OR DE "Minimal Brain Dysfunction" | 20,563 |
| S16 | S7 OR S8 OR S9 OR S10 OR S11 OR S12 OR S13 OR S14 | 527,086 |
| S15 | S5 OR S6 | 430,689 |
| S14 | TI ( ((specially-designed or assistive or adaptive) n2 (technolog* or equipment or product*)) ) OR AB ( ((specially-designed or assistive or adaptive) n2 (technolog* or equipment or product*)) ) OR TI ( ((staff* or personnel* or workforce*) n2 (train* or practic* or instruct*)) ) OR AB ( ((staff* or personnel* or workforce*) n2 (train* or practic* or instruct*)) ) OR TI ( (therap* n2 (social club* or support group* or organi?ation*)) ) OR AB ( (therap* n2 (social club* or support group* or organi?ation*)) ) OR TI ( ((peer* or group* or famil* or friend* or caregiver*) n2 (support* or assistance or aid)) ) OR AB ( ((peer* or group* or famil* or friend* or caregiver*) n2 (support* or assistance or aid)) ) OR TI ( (social n2 (activit* or gathering* or factor*)) ) OR AB ( (social n2 (activit* or gathering* or factor*)) ) OR TI ( ((social or societ* or communit*) n3 (condition* or environment* or context* or attitude* or inclus* or support*)) ) OR AB ( ((social or societ* or communit*) n3 (condition* or environment* or context* or attitude* or inclus* or support*)) ) | 90,511 |
| S13 | (((((((DE "Environmental Standards") OR (DE "Physical Environment")) OR (DE "Access to Health Care")) OR (DE "Attitudes toward Disabilities")) AND (DE "Social Discrimination" OR DE "Educational Discrimination" OR DE "Normalization (Disabilities)" OR DE "Social Bias" OR DE "Gender Bias" OR DE "Racism")) OR (DE "Public Opinion")) OR (DE "Labeling (of Persons)")) OR (DE "Stereotypes") | 15,434 |
| S12 | (((DE "Volunteers" OR DE "Student Volunteers" OR DE "Volunteer Training") AND (DE "Workplace Learning" OR DE "Occupational Safety and Health" OR DE "Facilities Management")) AND (DE "Community Involvement" OR DE "Community Planning" OR DE "Community Programs" OR DE "Community Resources" OR DE "Community Responsibility")) AND (DE "Legislation" OR DE "Civil Rights Legislation" OR DE "Educational Legislation" OR DE "Federal Legislation" OR DE "Labor Legislation" OR DE "State Legislation") | 11,436 |
| S11 | TI ( built environment* or sidewalk* or road* or street* or ramp* or transportation or paratransit or para transit or access* or aware* or attitude* or perception* or stereotyp* or discriminat* or stigma* or acceptance or inclusi* ) OR AB ( built environment* or sidewalk* or road* or street* or ramp* or transportation or paratransit or para transit or access* or aware* or attitude* or perception* or stereotyp* or discriminat* or stigma* or acceptance or inclusi* ) | 410,260 |
| S10 | TI ( ((environment* or facility or facilities or building* or city or cities or urban) n2 (design* or construct* or plan* or configur*)) ) OR AB ( ((environment* or facility or facilities or building* or city or cities or urban) n2 (design* or construct* or plan* or configur*)) ) | 12,548 |
| S9 | TI ( ((government* or legislat* or provinc* or municip* or federal* or national or organization* or organisation* or "not for profit" or "for profit" or nonprofit or non profit or association*) n2 (support* or assist* or policy or policies)) ) OR AB ( ((government* or legislat* or provinc* or municip* or federal* or national or organization* or organisation* or "not for profit" or "for profit" or nonprofit or non profit or association*) n2 (support* or assist* or policy or policies)) ) | 28,323 |
| S8 | TI (work* n2 remote*) OR AB (work* n2 remote*) | 324 |
| S7 | TI ( (communit* adj2 (service* or support* or integration* or factor* or resource* or system* or policy or policies or standard* or program* or assist* or design*)) ) OR AB ( (communit* adj2 (service* or support* or integration* or factor* or resource* or system* or policy or policies or standard* or program* or assist* or design*)) ) OR TI ( volunteer* or telework* or "work from home" or workplace* or work place* or work environment* or work space* or workspace* ) OR AB ( volunteer* or telework* or "work from home" or workplace* or work place* or work environment* or work space* or workspace* ) | 36,146 |
| S6 | TI ( employ* or work force* or workforce* or work or job* or occupation* or labo?r or re-employ* or reemploy* or unemploy* or un-employ* or vocation*) ) OR AB ( employ* or work force* or workforce* or work or job* or occupation* or labo?r or re-employ* or reemploy* or unemploy* or un-employ* or vocation*) ) | 427,926 |
| S5 | ((DE "Employment" OR DE "Multiple Employment" OR DE "Part Time Employment" OR DE "Seasonal Employment" OR DE "Student Employment" OR DE "Youth Employment") OR (DE "Supported Employment")) OR (DE "Careers") | 16,186 |
| S4 | S1 OR S2 OR S3 | 199,763 |
| S3 | TI ( (youth* or adolescen* or teen*) ) OR AB ( (youth* or adolescen* or teen*) ) OR TI ( (young* n2 (people* or adult*)) ) OR AB ( (young* n2 (people* or adult*)) ) | 126,093 |
| S2 | DE "Youth" | 7,229 |
| S1 | DE "High School Students" OR DE "Secondary School Students" OR DE "Adolescents" OR DE "High School Graduates" | 128,975 |

Bottom of Form

| Database | Sociological Abstracts |
| --- | --- |
| Interface | ProQuest |
| Date | May 2^nd^ 2024 |
| Limits | Date: 2008-Current |
| Number of Results | 379 |

((MAINSUBJECT.EXACT("Adolescents") OR MAINSUBJECT.EXACT("Young adults") OR summary(((youth* OR adolescen* OR teen*))) OR summary((young* NEAR/2 (people* OR adult*)))) AND (MAINSUBJECT.EXACT.EXPLODE("Employment") OR abstract((volunteer* OR telework* OR "work from home") OR ((workplace* OR work place* OR work environment* OR work space* OR workspace*)) OR ((employ* OR work force* OR workforce* OR work OR job* OR occupation* OR labo?r OR re-employ* OR reemploy* OR unemploy* OR un-employ* OR vocation*)) OR ((work* NEAR/2 remote*))) OR subject((volunteer* OR telework* OR "work from home") OR ((workplace* OR work place* OR work environment* OR work space* OR workspace*)) OR ((employ* OR work force* OR workforce* OR work OR job* OR occupation* OR labo?r OR re-employ* OR reemploy* OR unemploy* OR un-employ* OR vocation*)) OR ((communit* NEAR/2 (service* OR support* OR integration* OR factor* OR resource* OR system* OR policy OR policies OR standard* OR program* OR assist* OR design*))) OR ((work* NEAR/2 remote*))) OR title((volunteer* OR telework* OR "work from home") OR ((workplace* OR work place* OR work environment* OR work space* OR workspace*)) OR ((employ* OR work force* OR workforce* OR work OR job* OR occupation* OR labo?r OR re-employ* OR reemploy* OR unemploy* OR un-employ* OR vocation*)) OR ((communit* NEAR/2 (service* OR support* OR integration* OR factor* OR resource* OR system* OR policy OR policies OR standard* OR program* OR assist* OR design*))) OR ((work* NEAR/2 remote*)))) AND (abstract((disabilit* OR disabled OR impaired OR impairment* OR handicap*)) OR title((disabilit* OR disabled OR impaired OR impairment* OR handicap*)) OR abstract((deaf* OR "hard of hearing" OR blind* OR autism OR autistic OR asperger* OR chronic brain injury OR cerebral palsy OR spastic diplegia OR little disease OR little's disease OR wheelchair* OR mobility device* OR mobility scooter* OR mobility aid*) OR ((hearing OR audit*) NEAR/2 (impair* OR handicap*)) OR ((visual OR ocular OR vision) NEAR/2 (handicap* OR impair*))) OR title((deaf* OR "hard of hearing" OR blind* OR autism OR autistic OR asperger* OR chronic brain injury OR cerebral palsy OR spastic diplegia OR little disease OR little's disease OR wheelchair* OR mobility device* OR mobility scooter* OR mobility aid*) OR ((hearing OR audit*) NEAR/2 (impair* OR handicap*)) OR ((visual OR ocular OR vision) NEAR/2 (handicap* OR impair*)))) AND ((MAINSUBJECT.EXACT("Built environment") OR MAINSUBJECT.EXACT.EXPLODE("Facilities") OR (MAINSUBJECT.EXACT("Urban development") OR MAINSUBJECT.EXACT("Public transportation") OR MAINSUBJECT.EXACT("Community development") OR MAINSUBJECT.EXACT("Urban conditions")) OR (MAINSUBJECT.EXACT("Discrimination") OR MAINSUBJECT.EXACT("Access") OR MAINSUBJECT.EXACT("Equality") OR MAINSUBJECT.EXACT("Opportunities") OR MAINSUBJECT.EXACT("Barriers") OR MAINSUBJECT.EXACT("Constraints")) OR MAINSUBJECT.EXACT("Stereotypes") OR MAINSUBJECT.EXACT("Legislation") OR (MAINSUBJECT.EXACT("Community organizations") OR MAINSUBJECT.EXACT("Community involvement") OR MAINSUBJECT.EXACT("Community services")) OR MAINSUBJECT.EXACT("Volunteers") OR MAINSUBJECT.EXACT("Public opinion")) OR (abstract((((environment* OR facility OR facilities OR building* OR city OR cities OR urban) NEAR/2 (design* OR construct* OR plan* OR configur*)))) OR title((((environment* OR facility OR facilities OR building* OR city OR cities OR urban) NEAR/2 (design* OR construct* OR plan* OR configur*))))) OR (abstract((built environment* OR sidewalk* OR road* OR street* OR ramp* OR transportation OR paratransit OR para transit OR access* OR aware* OR attitude* OR perception* OR stereotyp* OR discriminat* OR stigma* OR acceptance OR inclusi*)) OR title((built environment* OR sidewalk* OR road* OR street* OR ramp* OR transportation OR paratransit OR para transit OR access* OR aware* OR attitude* OR perception* OR stereotyp* OR discriminat* OR stigma* OR acceptance OR inclusi*))) OR (abstract((((specially-designed OR assistive OR adaptive) NEAR/2 (technolog* OR equipment OR product*)))) OR title((((specially-designed OR assistive OR adaptive) NEAR/2 (technolog* OR equipment OR product*))))) OR (abstract((((staff* OR personnel* OR workforce*) NEAR/2 (train* OR practic* OR instruct*)))) OR title((((staff* OR personnel* OR workforce*) NEAR/2 (train* OR practic* OR instruct*))))) OR (abstract(((therap* NEAR/2 ("social club*" OR "support group*" OR organi?ation*)))) OR title(((therap* NEAR/2 ("social club*" OR "support group*" OR organi?ation*))))) OR (abstract((((peer* OR group* OR famil* OR friend* OR caregiver*) NEAR/2 (support* OR assistance OR aid)))) OR title((((peer* OR group* OR famil* OR friend* OR caregiver*) NEAR/2 (support* OR assistance OR aid))))) OR (abstract(((social NEAR/2 (activit* OR gathering* OR factor*)))) OR title(((social NEAR/2 (activit* OR gathering* OR factor*))))) OR (abstract((((social OR societ* OR communit* OR public) NEAR/3 (condition* OR environment* OR context* OR attitude* OR inclus* OR support* OR opinion*)))) OR title((((social OR societ* OR communit* OR public) NEAR/3 (condition* OR environment* OR context* OR attitude* OR inclus* OR support* OR opinion*))))) OR (abstract(((government* OR legislat* OR provinc* OR municip* OR federal* OR national* OR organization* OR organization* OR "not for profit" OR non-profit OR "non profit" OR for profit OR association*)) AND ((support* OR assist* OR policy OR policies))) OR title(((government* OR legislat* OR provinc* OR municip* OR federal* OR national* OR organization* OR organization* OR "not for profit" OR non-profit OR "non profit" OR for profit OR association*)) AND ((support* OR assist* OR policy OR policies)))))) AND pd(20080101-20241231)

| Database | Canadian Research Index |
| --- | --- |
| Interface | ProQuest |
| Date | April 25^th^ 2024 |
| Limits | Date: 2008-2024 |
| Number of Results | 24 |

Set#: S1

Searched for: ((youth* OR adolescen* OR teen* )) OR ((young* NEAR/2 (people* OR adult*)))

Databases: Canadian Research Index

Results: 3759

Set#: S4

Searched for: (disabilit* or disabled or impaired or impairment* or handicap*) OR ((deaf* OR "hard of hearing" OR blind* OR autism OR autistic OR asperger* OR chronic brain injury OR cerebral palsy OR spastic diplegia OR little disease OR little's disease OR wheelchair* OR mobility device* OR mobility scooter* OR mobility aid*) OR ((hearing OR audit*) NEAR/2 (impair* OR handicap*)) OR ((visual OR ocular OR vision) NEAR/2 (handicap* OR impair*)))

Databases: Canadian Research Index

Results: 5013

Set#: S6

Searched for: (volunteer* or telework* or "work from home") OR ((workplace* or work place* or work environment* or work space* or workspace*)) OR ((employ* or work force* or workforce* or work or job* or occupation* or labo?r or re-employ* or reemploy* or unemploy* or un-employ* or vocation*)) OR ((work* NEAR/2 remote*))

Databases: Canadian Research Index

Results: 60790

Set#: S7

Searched for: [S1] AND [S4] AND [S6]

Databases: Canadian Research Index

These databases are searched for part of your query.

Results: 111

Set#: S8

Searched for: ([S1] AND [S4] AND [S6]) AND pd(20080101-20241231)

Databases: Canadian Research Index

These databases are searched for part of your query.

Results: 24

| Database | Policy Commons |
| --- | --- |
| Interface | Coherent Digital |
| Date | April 25^th^ 2024 |
| Limits | Date: 2008-Current |
| Number of Results | 1007 |

summary:(youth OR adolesc* OR teen* OR young adult* OR young people OR young person) AND summary:(disab* OR deaf OR blind OR autis* OR asperger* OR wheelchair* OR mobility aid* OR cerebral palsy) AND summary:(employ* OR workforce* OR job OR jobs OR labor OR labour OR occupation* OR career* OR vocation*)

**
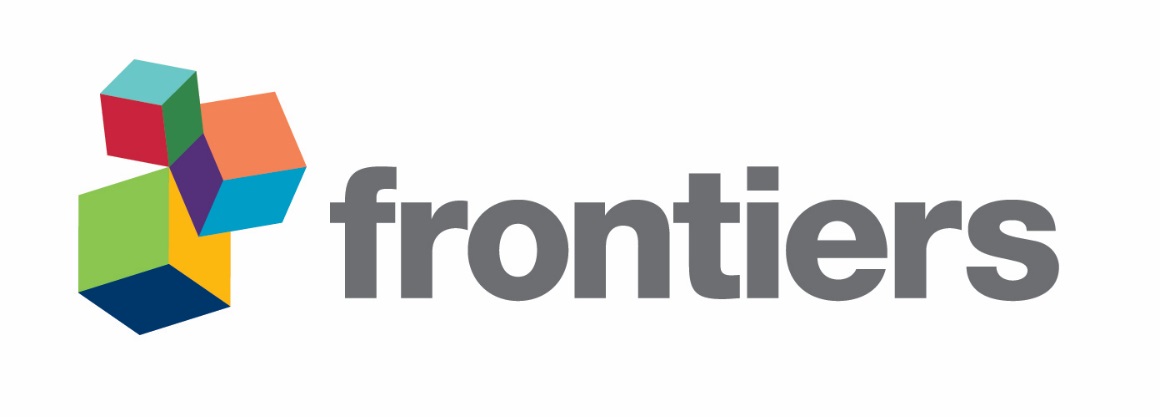
**
